# Sluggish post-garnet transformation controls slab stagnation at the uppermost lower mantle

**DOI:** 10.1038/s41467-026-72495-5

**Published:** 2026-04-23

**Authors:** Yongqiang Shen, Jianfeng Yang, Liang Zhao

**Affiliations:** 1 State Key Laboratory of Lithospheric and Environmental Coevolution, Institute of Geology and Geophysics, Chinese Academy of Sciences, Beijing, China; 2 College of Earth and Planetary Sciences, University of Chinese Academy of Sciences, Beijing, China; 3 Key Laboratory of Deep Petroleum Intelligent Exploration and Development, Institute of Geology and Geophysics, Chinese Academy of Sciences, Beijing, China

**Keywords:** Geodynamics, Geophysics

## Abstract

The behavior of subducted slabs in the deep mantle is crucial for understanding Earth’s evolution and mantle dynamics. Seismic observations reveal widespread slab stagnation at around 1000 km, yet no major phase transitions have been found at that depth, leaving the underlying mechanisms enigmatic. Here we show that sluggish kinetics of the post-garnet transformation could induce a critical density deficit within subducting slabs. This deficit sustains metastable garnet over tens of millions of years, effectively stalling slabs at the uppermost lower mantle. Our results reveal that slab stagnation at this depth is transient and inherently tied to delayed phase transformation kinetics, consistent with geophysical inferences. This offers an alternative explanation for slab stagnation in a pyrolitic mantle without requiring long-standing rheological or chemical explanations alone. These findings highlight a critical, yet underexplored, role of phase transformation kinetics in slab behavior and deep mantle dynamics.

## Abstract (web summary)

Sluggish post-garnet kinetics persist metastable garnet in subducting slabs, creating a density deficit that stalls slabs at ~1000 km depth transiently, explaining seismic observations without requiring mantle heterogeneity.

## Introduction

**Fig. 1:**
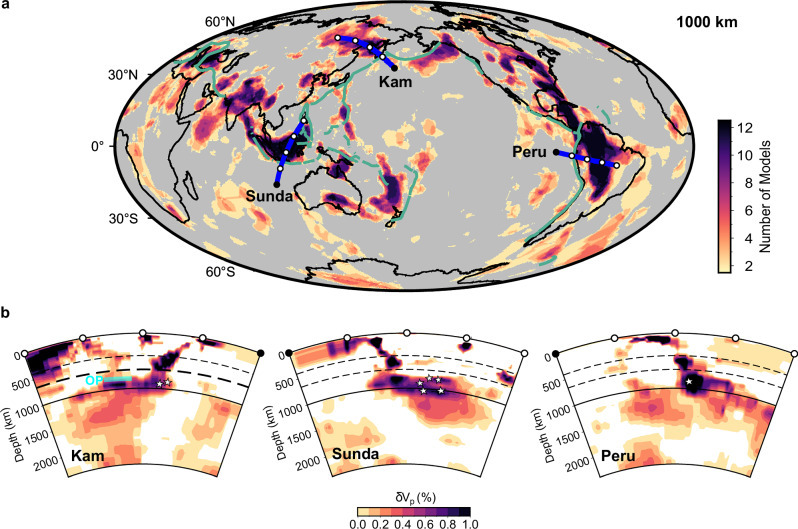
Seismic evidence of slab stagnation in the uppermost lower mantle. **a** High-velocity vote maps for P-wave models at 1,000 km depth. The vote map data are derived from the SubMachine website^64^. Green lines are tracks of present subduction zones, while blue lines are positions of tomography cross-sections. **b** Cross sections of the UUP07 model^65^ at the Kamchatka (Kam), Sunda, and Peru subduction zones. The cyan bar denoted by OP in the cross-section of the Kamchatka subduction zone represents a possible thick oceanic plateau identified by ref. ^25^. The white stars denote the average locations of S-to-P wave scatterers identified by ref. ^26^.

The dynamics of subducted slabs in the deep mantle critically influence mantle convection patterns and mass exchange between different mantle reservoirs. These dynamics are primarily governed by the density and viscosity contrasts between slabs and the surrounding mantle. A well-documented example is the density increase associated with the post-spinel transformation (ringwoodite to ferropericlase and bridgmanite) at ~660 km depth, which often causes slab stagnation at the mantle transition zone^1,2^, potentially stratifying the mantle convection regime. Intriguingly, seismic imaging reveals another stagnation horizon for subducted slabs at ~1000 km depth^3^ (Fig. 1), yet no major phase transition is known at this depth^4^. The mechanisms responsible for this deeper stagnation phenomenon remain poorly understood and constitute an important open question in deep Earth dynamics.

Prevailing hypotheses for this deeper stagnation have primarily invoked changes in mantle composition or viscosity. For instance, slab stagnation could be induced by basaltic enrichment in the lower mantle^5^ or mid-mantle viscosity jump^6–9^. These mechanisms provide viable explanations and highlight the potential roles of chemical and rheological heterogeneity. Nevertheless, the general applicability of these models is subject to ongoing debate. While studies support pervasive basaltic heterogeneities^10^, others provide evidence for a predominantly pyrolitic composition of the bulk lower mantle^11^. Moreover, previous numerical models suggest that slab stagnation may be more strongly controlled by crust strength than by the viscosity contrast itself^12^. Furthermore, recent studies propose that dislocation creep or climb may dominate under high-strain conditions near subducted slabs^13–16^, a deformation mechanism that would reduce local viscosity and thereby facilitate, rather than impede, slab penetration.

**Fig. 2:**
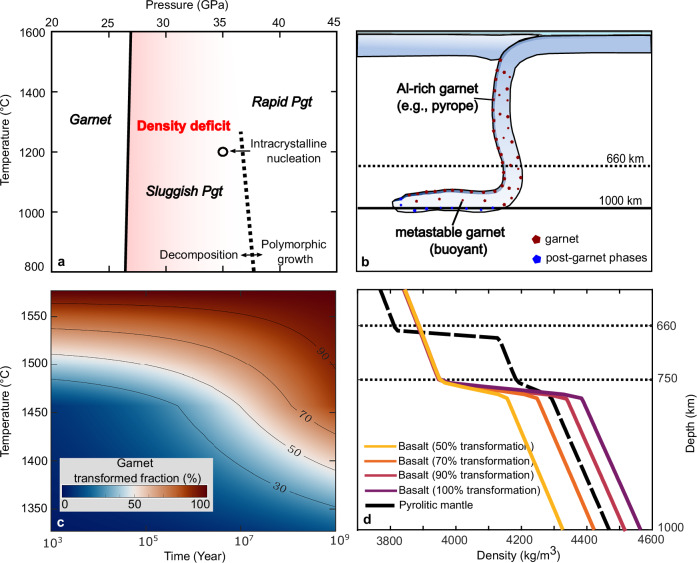
Slow kinetics of post-garnet transformation (pgt) and its impact on basalt density. **a** Boundaries of nucleation and growth mechanisms for the post-garnet transformation, modified after ref. ^23^. See Supplementary Fig. 1 for the post-garnet transition boundary used in this study. **b** Schematic diagram showing the residue metastable garnet in the slab and its contribution to slab stagnation in the uppermost lower mantle. **c** Transformed fractions of garnet as a function of ambient temperature and time interval since the initiation of post-garnet transition. The diagram is obtained based on the growth kinetics of post-garnet assemblages determined by ref. ^22^, and the garnet grain size is set to be 4 mm. **d** Density profiles of basalt across the uppermost lower mantle with varying transformation fractions of garnet.

Beyond these chemical and rheological explanations, a potentially critical yet neglected factor lies in the kinetics of phase transformation. Laboratory experiments suggest that sluggish kinetics, such as post-garnet transition, allow low-density metastable phases to persist within cold slabs to greater depths^17^. The post-garnet transformation, which under equilibrium conditions completes at ~720–760 km depth (~25–28 GPa)^18–20^, exhibits slow growth kinetics^21–23^. As a result, garnet can survive metastably to depths of ~950–1000 km (~35–38 GPa, Fig. 2) before fully decomposing. The presence of metastable garnet may create a density deficit relative to the surrounding mantle^22^. Such metastability has been implicated in shallow stagnation (e.g., pyroxene-garnet transition)^24^, but its role in the lower mantle remains untested.

Here, we integrate thermomechanical models with experimentally constrained post-garnet transformation kinetics to resolve this paradox. We demonstrate that metastable garnet in the subducted slab could induce slab stagnancy at the uppermost lower mantle for tens of millions of years, independent of mantle composition and viscosity. By quantifying the roles of garnet content, slab dip, and thermal age, we reconcile global seismic observations of transient slab stagnation within a pyrolitic mantle.

## Results

### Model setup

To investigate slab stagnation mechanisms in the lower mantle, we developed two-dimensional thermomechanical models with a whole mantle scale, explicitly integrating post-garnet transformation kinetics. Our models incorporate experimentally determined kinetic constraints for the post-garnet transformation^22^ (Fig. 2), which account for the effect of temperature, garnet grain size, and growth timescales of post-garnet phases (Supplementary Fig. 2). We use the flow laws of Nabarro-Herring creep and pure climb creep to describe the deformation of the lower mantle (Supplementary Figs. 3 and 5b). To resolve the thermally controlled phase transformation, we implemented a temperature-pressure-composition-dependent thermal conductivity, enabling realistic slab-mantle thermal exchange (Supplementary Fig. 6d). The subducting slab comprises three distinct layers: the oceanic crust, the harzburgitic mantle, and the lherzolitic mantle. The garnet content, calibrated from natural and laboratory constraints, in each slab component before the initiation of post-garnet transformation is set to be 25–50% in the oceanic crust, 0–10% in the harzburgitic mantle, and 10–15% in the lherzolitic mantle, respectively (Supplementary Fig. 5). By contrasting two end-member scenarios: equilibrium (instantaneous phase change) versus non-equilibrium (kinetically delayed) transformation, we systematically evaluate how slab geometry, thermal age, Clapeyron slope of the post-garnet transition, and lower mantle viscosity jump govern stagnation versus penetration behavior.

### Equilibrium and non-equilibrium of post-garnet phase transformation

**Fig. 3:**
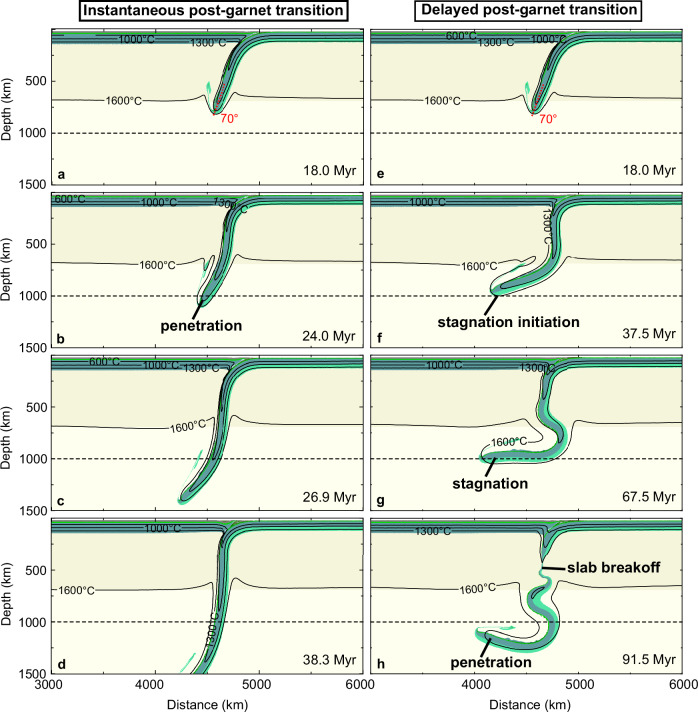
Model evolution of composition fields for two cases of post-garnet transformation. **a**–**d** Rapid post-garnet transformation (equilibrium) case. **e**–**h** Delayed post-garnet transformation (non-equilibrium) case. The subduction dip angles of which slabs enter the lower mantle are indicated in (**a** and **e**). The 1000 km depth is indicated with a dashed line.

**Fig. 4:**
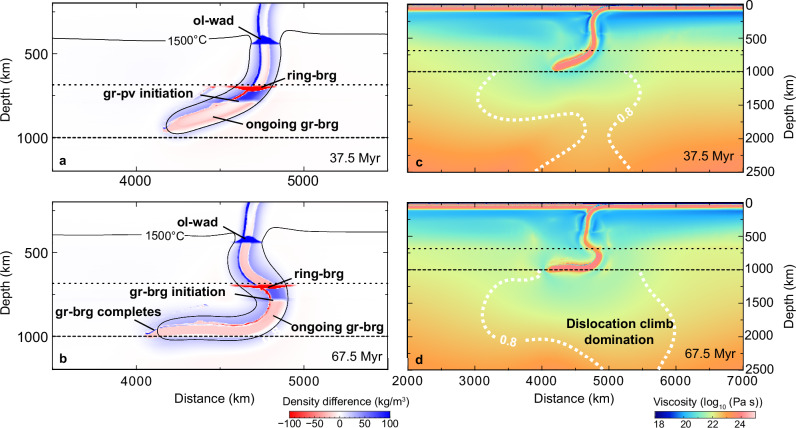
Density difference and viscosity fields of the model of non-equilibrium transformation. **a,** **b** Distribution of density difference. The differences are relative to the density profile at 2000 km. **c,** **d** Viscosity field. The white dashed lines enclose the lower mantle region where the pure climb creep ratio exceeds 0.8. The pure climb creep ratio is defined by the ratio of pure climb creep viscosity to the total viscosity. The black dashed lines are the 660-km and 1000-km depths. Abbreviations: olivine (ol), wadsleyite (wad), ringwoodite (ring), perovskite (pv), garnet (gr).

Under equilibrium conditions, instantaneous post-garnet transformation enables rapid slab penetration through the uppermost lower mantle (660–1000 km) at a rate of 3–4 cm⋅yr^-1^, with no stagnation. In contrast, non-equilibrium transformation preserves metastable garnet in cold slab interiors, inducing stagnation. For the reference model (35% garnet in oceanic crust, 6% in harzburgitic mantle, 15% in lherzolitic mantle; 4 mm grain size), the slab decelerates upon crossing the 660 km discontinuity, bending forward and flattening above the 1000 km depth (Fig. 3f, g). Slab stagnation initiates at ~37 Myr and persists until ~70 Myr, during which slab material accumulates, triggering backward buckling before eventual penetration into the deep lower mantle (Fig. 3h). Stagnant slabs exhibit dramatically reduced sinking velocities (<0.4 cm⋅yr^-1^ on average) and long-distance stagnation (~900 km), sustained by a density deficit from metastable garnet (Fig. 4a, b). This deficit diminishes as temperatures and pressures rise, which drives the late-stage transformation, enabling a renewed sinking once net negative buoyancy is restored. As the slab descends into the lower mantle, the mantle surrounding the slab is significantly weakened (Fig. 4c, d, and Supplementary Fig. 6) due to the dominance of pure climb creep in these regions. This favors the slab subduction into the deep lower mantle.

### Kinetic controls on stagnation modes

**Fig. 5:**
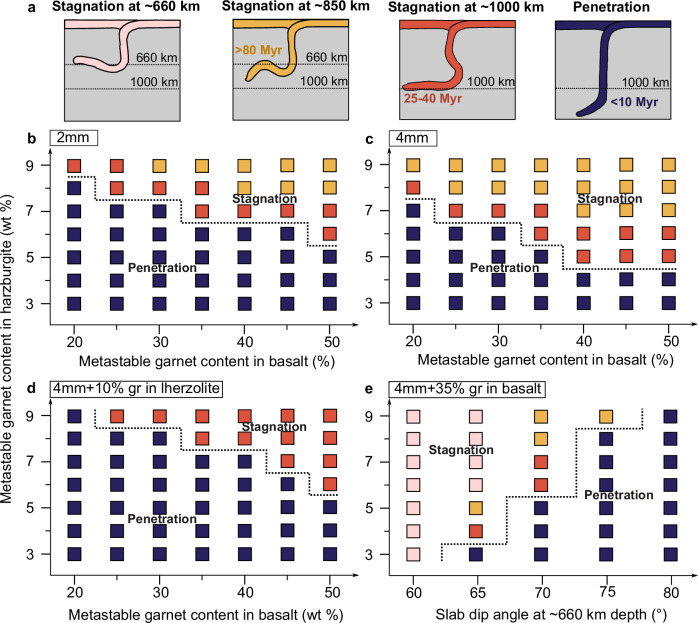
Model tests on metastable garnet content, grain size, and slab geometry. **a** A summary of typical modes of slab behavior in the lower mantle. **b**, **c**Model tests on metastable garnet contents in basaltic crust and harzburgitic lithosphere for different grain sizes (2 mm, 4 mm). **d** Model tests on metastable garnet content in basaltic crust and harzburgitic lithosphere for 10% garnet content in the lherzolitic mantle. **e** Model tests on slab geometry. The slab geometry refers to the dip angle of the slab at the base of the mantle transition zone (e.g., Fig. 3a). Abbreviation: garnet (gr).

Garnet grain size and content critically regulate post-garnet transformation efficiency and slab buoyancy, respectively. Larger grains (e.g., >4 mm) prolong metastability, amplifying density deficit (Supplementary Fig. 2). Systematic model tests reveal three endmember modes of slab behavior in the uppermost lower mantle (Fig. 5 and Supplementary Fig. 8): penetration, 1000-km-stagnation, and 850-km-stagnation. The penetration mode typically occurs for a low garnet content (<5% for the harzburgite and <20% for the basalt) and small grain size (<2 mm). While an intermediate garnet content (5–8% for the harzburgite and 20–50% for the basalt) and grain size (2–5 mm) induce transient stagnation for 25–40 Myr at ~1000 km. A high garnet content (>8% for the harzburgite and >50% for the basalt) and large grain size (>5 mm) tend to induce stagnation at ~850 km for >80 Myr, with intense slab buckling resulting from enhanced buoyancy (Supplementary Fig. 8). The slab behavior is more sensitive to garnet content in the harzburgitic and lherzolitic mantle due to their greater thickness.

### Effects of slab geometry and thermal age

Building upon our stagnation reference model, we systematically investigate how slab geometry (60 °–80 ° dip angles at the base of the mantle transition zone) and thermal age (40–130 Myr) influence stagnation. Model results reveal a clear dependence on the dip angle. Steeper slabs (75 °–80°) favor rapid penetration (Fig. 5b and Supplementary Fig. 9), while shallower angles (<70 °) induce stagnation, with stagnation depth progressively decreasing from 800–1000 km to <660 km as the dip decreases. Shallow-dipping slabs (e.g., 65 °) require less metastable garnet content for 1000 km stagnation (Supplementary Fig. 10), highlighting the critical role of low-angle subduction on stagnation. Slab thermal age exerts additional control on stagnation behavior: younger slabs (<50 Myr) typically stagnate around 850 km (Supplementary Fig. 10), whereas older slabs (>70 Myr) favor 1000 km stagnation. Despite elevated interior temperatures in younger slabs, the 1600 K threshold for post-garnet transformation^22^ limits the effects of slab thermal age on the phase transition. Critically, slab geometry outweighs thermal age in controlling stagnation (Supplementary Fig. 10): steep-dipping slabs penetrate even at younger ages due to (1) rapid completion of post-garnet transformation and (2) enhanced bending resistance during high-angle subduction.

### Reassessing stagnation mechanisms of viscosity jump

To assess the potential influence of a mid-mantle viscosity jump, we model a scenario with a ~ 1-order-of-magnitude increase in viscosity at 1000 km depth, mimicking the effect of bridgmanite grain growth^8^ (Supplementary Fig. 11). The viscosity jump, however, fails to impede slab penetration. The grain-size-independent dislocation creep (Supplementary Fig. 11) dominates the deformation in the slab’s periphery, reducing local mantle viscosity by 1–2 orders (Supplementary Fig. 11). This localized weakening facilitates, rather than hinders, slab descent. Notably, the mantle viscosity adjacent to the subducting slab in our model is ~1 order of magnitude higher than that proposed for a ferropericlase strengthening^9^ (Supplementary Fig. 11d). This implies that the ferropericlase-induced viscosity jump may be unlikely to be the primary cause of slab stagnation at this depth.

### Effect of phase transitions near the mantle transition zone

We conducted additional tests to evaluate the influence of major phase transitions near the mantle transition zone (410–750 km) on our proposed mechanism (Supplementary Fig. 12). Model results suggest: (1) The olivine-wadsleyite and wadsleyite-ringwoodite transitions have a minor influence, slightly reducing the stagnation duration without altering the stagnation mode at ~1000 km. (2) The Clapeyron slope of the ringwoodite-bridgmanite transition is a key control at 660 km. A shallow slope (−0.1 MPa⋅K^-1^) favors slab penetration. In contrast, a steeper Clapeyron slope (−3.0 MPa⋅K^-1^) promotes 660-km stagnation. (3) The akimotoite-bridgmanite transition, despite its strong negative Clapeyron slope, has a small effect on slab behavior in the lower mantle due to the low modal proportion of akimotoite in the subducting slab (Supplementary Fig. 12c). (4) A negative Clapeyron slope of post-garnet transition favors shallower stagnation mode due to the late post-garnet transition in the cold slab (Supplementary Fig. 13). However, this negative-Clapeyron-slope transition alone is insufficient to cause slab stagnation without residue metastable garnet.

## Discussion

Our model results demonstrate that sluggish post-garnet transformation kinetics induce transient slab stagnation in the uppermost lower mantle (660–1000 km) over tens of millions of years. This process hinges on the retention of metastable garnet within subducting slabs, a mechanism supported by seismic observations. For example, the Kamchatka subduction zone exhibits a stagnant slab at 800–835 km depth overlain by a ~ 35-km-thick oceanic plateau^25^, where metastable garnet in the basaltic crust was suggested to survive to a depth of 810 km due to the low thermal gradient of the oceanic plateau. Density calculations indicated that a ~ 10-km-thick metastable garnet layer can neutralize slab buoyancy^25^. Beyond the Kamchatka, pervasive seismic scatterers and discontinuities within the interior of stagnant slabs in the uppermost lower mantle^26^ (Sunda, Nazca, and Peru slabs), and lower-mantle-penetrating slabs^26–28^ (Mariana, Alaska, and Philippine slabs), align spatially with predicted garnet-rich regions.

**Fig. 6:**
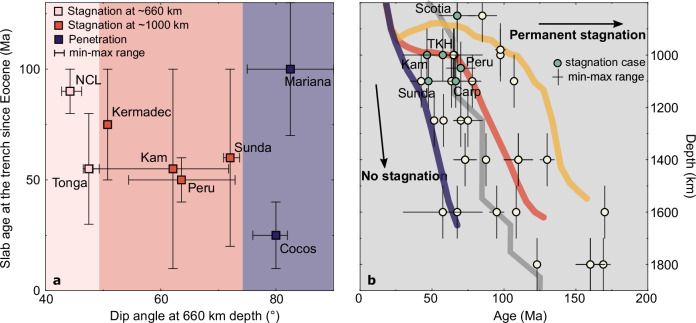
Observation suggests the role of slab geometry in slab stagnation and a temporary feature of slab stagnation. **a** Slab age versus dip angle at the base of the mantle transition zone. The slab dip angles at the Sunda, Kamchatka (Kam), Peru, Kermadec, Tonga, and North Chile (NCL) subduction zones are derived from ref. ^66^, while those of the Cocos slab and Mariana slab are estimated based on ref. ^67^ and ref. ^68^, respectively. The data points denoted by color-filled rectangles are average values. **b** Depth-age relations of tomographically imaged remnant slabs between 800 and 1800 km depth. The depth-age relations are derived from ref. ^29^. Green-filled circles represent stagnation cases, while white-filled circles are non-stagnation cases. The three color-coded bold lines represent characteristic depth-time relationships of three slab behavior modes: penetration (dark blue), 800-km-stagnation, and 1000-km-stagnation. The thick grey line derived from ref. ^69^ is the best-fit time-depth curve through averaging ten tomography models. Subduction zone or slab name abbreviation: Kamchatka (Kam), Tonga-Kermadec-Hikurangi (TKH), Carpentaria (Carp).

Natural subduction systems exhibit diverse slab behaviors from deep penetration to stagnation. Our models reveal this variability through two controlling factors: (1) metastable garnet content and (2) slab dip angle at 500–660 km depth. Although the garnet content is difficult to estimate in each slab, the slab dip angle could be well resolved. Systematic analysis of natural slabs shows a clear correlation between dip angle and stagnation behavior (Fig. 6): Steeply dipping slabs (e.g., Mariana and Cocos slabs) typically penetrate the uppermost lower mantle; moderately dipping slabs (e.g., Sunda and Kamchatka slabs) often stagnate temporarily in the uppermost lower mantle; while shallow-dipping slabs (e.g., Nazca slab beneath North Chile and Tonga slab) generally stagnate at the mantle transition zone. This dip-angle dependence provides a plausible mechanism for explaining the global variability in lower-mantle slab behavior across different subduction zones.

The transient slab stagnation in our models is attributed to the progressive garnet transformation and to density equilibration of the slab with the ambient mantle. This aligns with seismic observations which show several stagnant slabs continuously extend to ~1500 km without prolonged arrest (e.g., Sunda slab, Nazca slab, and Kamchatka slab, Fig. 1). More importantly, the global compilation of seismic-imaged remnant slab ages and depths in the lower mantle (Fig. 6) shows generally linear age-depth relations without a sudden break or a wide distribution of age at a certain depth^29^. This further supports the ephemeral nature of slab stagnation in the uppermost lower mantle. Based on our models, we analyze the depth-time relations of slabs for different slab behavior modes (850-km-stagnation mode, 1000-km-stagnation mode, penetration mode; Fig. 6). Our predicted stagnation durations (25–80 Myr) reconcile with geophysical inferences of episodic slab stagnation in the uppermost lower mantle.

The long-standing puzzle of slab stagnation at the uppermost lower mantle has been primarily attributed to two distinct classes of mechanisms: chemical heterogeneity and increased mid-mantle viscosity. Evidence for chemical heterogeneity, often interpreted as the presence of recycled basaltic crust, is supported by widespread seismic scatterers and mineral physics constraints that require the presence of basaltic heterogeneity to match seismic observations^10,26,30^. Geodynamic models suggest that the enrichment of dense basaltic lithologies in the lower mantle may render slabs neutrally buoyant. Mid-mantle viscosity jumps, arising from mechanisms such as bridgmanite grain growth^8^, ferropericlase strengthening^9^, or a decrease in oxygen vacancy concentration^7^, have also been invoked as an alternative explanation. Our modeling results, however, indicate that such viscosity jumps may be insufficient to stagnate slabs on their own, especially when grain-size-insensitive dislocation climb dominates deformation in the high-stress periphery of the slab (Supplementary Fig. 11). Similarly, the viscosity increase predicted from ferropericlase strengthening is not large enough either to stagnate subducting slabs (Supplementary Fig. 11). Nonetheless, the precise physical mechanism responsible for a mid-mantle viscosity jump still remains unclear, and its potential effect on slab dynamics cannot be fully excluded. In this study, we demonstrate that sluggish post-garnet transformation kinetics provide a robust, alternative mechanism for transient slab stagnation within a predominantly pyrolitic mantle. This kinetic mechanism does not exclude the potential roles of chemical heterogeneity or rheological variations. Rather, it could operate independently or synergistically with them. For instance, a higher intrinsic garnet content in basaltic material would enhance the kinetic buoyancy effect. The relative importance of kinetics, composition, and rheology likely varies across subduction zones, controlled by factors such as slab age, dip angle, and hydration. Future studies that self-consistently integrate phase kinetics, compositional heterogeneity, and complex rheology will be crucial to fully unravel their combined control on deep slab dynamics.

Garnet grain size critically controls post-garnet transformation efficiency. Our chosen ranges (2–6 mm) are representative values observed in natural samples, though we acknowledge the uncertainty in this parameter and our assumption of a constant grain size. In nature, both deformation-induced grain reduction (accelerating transformation) and thermal-induced grain growth (prolonging metastability) could alter grain size during subduction^31^. For instance, slab heating upon encountering the hot mantle accelerates garnet growth, thereby slowing the post-garnet transformation and promoting stagnation. Conversely, grain reduction in the slab bending area^31^ accelerates the transformation, thereby facilitating slab penetration. However, the robustness of our stagnation results across the tested grain-size range confirms the validity of the kinetic mechanism. Future work constraining the grain size evolution will refine the slab dynamics and stagnation timescales. The presence of water may further modulate the stagnation behavior. As water is known to catalyze mineral reactions, its presence in the slab would be expected to accelerate the kinetics of post-garnet transformation. We could speculate that a severely dehydrated slab would experience even slower transformation kinetics, likely enhancing metastability and prolonging stagnation. In contrast, a slab with local hydrous garnet could undergo accelerated transformation, potentially reducing the stagnant effect. Therefore, the variable hydration states of subducting slabs may contribute to the global spectrum of observed slab behaviors. Furthermore, our findings are based on 2D thermomechanical models, while subduction is an inherently 3D process. However, the proposed mechanism of kinetically induced buoyancy is an intrinsic property of the slab, which can be fundamentally captured in our models. This approach is particularly relevant for the mid-sections of major 1000-km-stagnation subduction zones (e.g., Sunda, Peru; Fig. 1) where the mantle flow is predominantly poloidal. We acknowledge that 3D processes such as toroidal flow around slab edges and trench retreat are not produced here. These effects can alter the slab’s thermal structure and dip angle. Notably, our systematic tests on slab dip angle show that shallower dips, a common consequence of 3D rollback, promote stagnation at 660 km or 850 km. This is consistent with observations near slab edges like Kamchatka. Therefore, while the specific expression of stagnation may be modulated by 3D geometry, the fundamental kinetic control we identify remains a widely applicable mechanism. Future 3D models will be valuable to explore the interaction between kinetic buoyancy and the complex flow patterns around the slab edges.

A foundational premise of our model is that post-garnet transformation is sufficiently sluggish to sustain metastability over geological timescales, which rests on experimental data; yet extrapolating this to millions of years of geological processes carries inherent uncertainty. Furthermore, natural garnet composition varies across slab lithologies (e.g., pyrope, grossular, almandite), which may lead to different transformation kinetics and equilibrium boundaries. Our use of pyropic garnet as a uniform proxy is a necessary simplification given the current lack of systematic kinetic data across the full compositional spectrum. Thus, while existing high-pressure experiments support the possibility of sluggish garnet transformation, the absolute timescale and composition-dependent behavior of metastability in Earth’s mantle are not yet uniquely determined. Our model quantifies the geodynamic consequences if such kinetics are operative. Further high-pressure, low-temperature experimental studies are needed to better constrain the kinetics of the post-garnet transformation and to evaluate the influence of garnet composition.

In summary, we highlight the critical role of phase transformation kinetics in controlling slab dynamics in the lower mantle. Similar kinetic effects have been documented in previous studies of the olivine-spinel transition^32^ and the pyroxene-garnet transition^24^, where metastable olivine or pyroxene persistence can induce slab stagnation in the mantle transition zone. While stagnant slabs temporarily reside in the uppermost lower mantle, they ultimately descend into the core-mantle boundary region and fuel mantle plumes, thereby participating in whole-mantle material recycling. The kinetic barriers in phase transformation may thus spatially and temporally modulate mass exchange between the upper and lower mantle reservoirs, ultimately maintaining Earth’s long-term geochemical and thermal equilibrium.

## Methods

### Governing equations

The thermomechanical numerical code I2VIS^33,34^, based on the finite difference method and marker-in-cell technique, is applied to solve the incompressible flow for the extended Boussinesq approximation (EBA). Three conservation equations are considered: mass, momentum, and energy conservation, expressed as:

1 $$\frac{\partial {v}_{i}}{\partial {x}_{i}}=0$$

2 $$\frac{\partial {\sigma }_{{ij}}}{\partial {x}_{i}}-\frac{\partial {P}_{i}}{\partial {x}_{i}}=-\rho {g}_{i}$$

3 $$\rho {C}_{p}\frac{{DT}}{{Dt}}=\frac{\partial }{\partial {x}_{i}}\left(k\frac{\partial T}{\partial {x}_{i}}\right)+{H}_{r}+{H}_{s}+{H}_{a}+{H}_{L}$$

where ${{\bf{v}}}$ is the velocity, ${{\boldsymbol{\sigma }}}$ is the deviatoric stress, $\rho$ is the density, ${{\bf{g}}}$ is the gravitational acceleration, ${C}_{p}$ is the heat capacity, $T$ is the temperature, $P$ is the total pressure, $k$ is the thermal conductivity and ${H}_{r}$, ${H}_{s}$, ${H}_{a}$ and ${H}_{L}$ are the radioactive, shear, adiabatic, and latent heat productions, respectively.

### Rock density and phase transitions

Rock density is calculated by $\rho \left(P,T\right)={\rho }_{0}\left[1-\alpha \left(T-{T}_{0}\right)\right]\left[1+\beta \left(P-{P}_{0}\right)\right]+F\Delta {\rho }_{{ph}}$, where ${\rho }_{0}$ is the reference density for *P*_*0*_ = 10^5 ^Pa and T_0_ = 298 K, $\alpha$ is the thermal expansion coefficient, and $\beta$ is the compressibility coefficient. $\Delta {\rho }_{{ph}}$ is the density variation induced by phase transformation, and *F* is the transformed volume fraction.

Our model employs the EBA, which solves the incompressible Stokes equations with a depth-dependent density profile, while incorporating the non-Boussinesq term, including adiabatic heating, shear heating, and latent heating. This formulation neglects dynamic volume changes associated with advection and compression but fully captures the buoyancy forces arising from thermal and compositional density anomalies. Although the density varies significantly with the whole mantle depth (Supplementary Fig. 6), the EBA is considered a robust approximation for studying large-scale mantle dynamics as the errors introduced are typically small compared to other model uncertainties for this class of problems^35^.

We consider eclogitization, coesite-stishovite and post-garnet transformation in the basaltic and gabbroic crust, and olivine-wadsleyite, ringwoodite-perovskite and post-garnet transformation in mantle rocks (Supplementary Fig. 1). The equilibrium post-garnet transition boundaries vary with garnet end-members (e.g., pyrope, grossular, almandine, majorite) and mantle compositions (e.g., pyrolite and harzburgite) (Supplementary Fig. 1). In simplicity, we use the phase boundary of basaltic crust as a proxy for all rock types and garnet end-members. This simplification is justified for three reasons: 1) The basaltic crust has the largest garnet content in the slab at the lower mantle conditions, and its transition boundary is closely aligned with those of pyrope and majorite in pyrolitic mantle at uppermost lower mantle conditions, making it a representative average. 2) The phase boundary of majorite in harzburgite is close to that of the ringwoodite-bridgmanite transition (Supplementary Fig. 1b), and its effect on density could be integrated with that of the ringwoodite-bridgmanite transition. 3) For other garnet end-members like grossular and almandine, they decompose at ~22–26 and ~19–21 GPa in equilibrium, respectively^36–38^. High-pressure experiments suggested slow kinetics of their decomposition reaction^36,37,39^, which allow them to survive in metastable phases up to pressures of 27–30 GPa. We thus assume the same post-garnet transition boundary for them as that of the basaltic crust in this study. This simplification facilitates our definition of a single parameter of garnet content, and also parameter test and discussion on garnet content. A recent study identified a varying slope of the post-garnet transformation with temperature based on experiments on pyrope (Mg_3_Al_2_Si_3_O_12_), with a negative Clapeyron slope at lower temperature, while a positive Clapeyron slope at high temperature^19^. Based on our reference model, we additionally explore the effect of this phase transition boundary (Supplementary Fig. 1b). The $\Delta {\rho }_{{ph}}$ of each transformation is according to refs. ^40^^,^^41^. The $\alpha$ and $\beta$ are constant in the upper mantle but linearly decrease in the lower mantle to approximate the density profile presented by ref. ^42^ (Table S1 and Supplementary Fig. 6).

### Kinetics of post-garnet transformation

We consider the kinetics of post-garnet transformation in our model. The post-garnet transformation does not complete once the phase boundary condition is satisfied but proceeds at a slow rate, leading to a non-equilibrium state of phase transformation and the existence of metastable garnet at pressures higher than the phase boundary condition. The kinetics of the post-garnet transformation are derived from ref. ^22^ in which the transformed volume *F* is a function of grain size (*d*), temperature (*T*), and growth time (*t*) (Supplementary Fig. 2; Equation A4). Ref. ^22^ presented *F*-*t* relations of post-garnet transformation at 1320 K, 1600 K, 1730 K, and 1850 K (Supplementary Fig. 2) through high temperature and pressure experiments. Here, we extend these temperatures to the whole temperature range (1,320–1,850 K) through linear interpolation. Supplementary Fig. 2 shows the result of *F* as a function of temperature and growth time for grain sizes of 2 mm and 6 mm. In addition to the temperature, grain size and growth time, we further account for enhanced transformation kinetics at 35–38 GPa (950–1000 km depth) due to the growth mechanism shift from decomposition to polymorphic growth^23^, assuming complete transformation (*F* = 1.0) when P-T conditions cross this threshold (dashed line in Fig. 2). The kinetics of the post-garnet transformation mentioned above is only considered within the cold slab in our model, since the high temperature of the lower mantle and long-term mantle convection facilitates a complete post-garnet transformation.

4 $$F=1-exp \left\{\frac{-2\times 3.35\gamma {t}^{\theta }}{d}\right\}$$

where $\gamma$ and $\theta$ are kinetic constants, *t* is the growth time of post-garnet phase which is counted from the moment when post-garnet transformation begins. The values of $\gamma$ and $\theta$ at temperatures of 1,600 K, 1,730 K, and 1,850 K are derived from ref. ^22^.

The grain size of garnet in natural eclogite and peridotite ranges from 1 to 10 mm^21,43–45^, and it may not vary significantly during subduction due to no transformations in garnet before entering the lower mantle^21^. It has a great effect on the kinetics of post-garnet transformation (Supplementary Fig. 2). The post-garnet transformation is more sluggish in garnet with a larger grain size (Supplementary Fig. 2).

### Rock rheology

Rock deformation follows visco-plastic flow laws. Viscous deformation is controlled by diffusion creep (${\eta }_{{diff}}$) and dislocation creep (${\eta }_{{disl}}$). The viscosity of the continental crust and oceanic crust is calculated based on the flow law of Ranalli^46^:

5 $${\eta }_{{diff}}=\frac{1}{2}{A}_{d}{\sigma }_{{crit}}^{1-n}exp \left(\frac{P{V}_{a}+{E}_{a}}{{RT}}\right)$$

6 $${\eta }_{{disl}}=\frac{1}{2}{A}_{d}^{\frac{1}{n}}{\dot{\varepsilon }}_{{II}}^{\frac{1-n}{n}}exp \left(\frac{P{V}_{a}+{E}_{a}}{{nRT}}\right)$$

where ${A}_{d}$ is the pre-exponential constant; $n$ is the stress/strain rate exponent; ${V}_{a}$ is the activation volume; ${E}_{a}$ is the activation energy; $R$ is the gas constant (8.314 J·K^-1^mol^-1^); ${\sigma }_{{crit}}$ (10^4^ Pa) is the transition stress from diffusion creep to dislocation creep;${\dot{{{\boldsymbol{\varepsilon }}}}}_{{{\boldsymbol{II}}}}$ is the second invariant of strain rate tensor.

The flow law of the upper mantle and the mantle transition zone follows that of ref. ^47^, which is calculated as:

7 $${\eta }_{{diff},\,{disl}}=\frac{1}{2}{({A}_{k})}^{\frac{-1}{n}}\mu {\left(\frac{d}{b}\right)}^{\frac{m}{n}}{\left({\dot{\varepsilon }}_{{II}}\right)}^{\frac{1-n}{n}}exp \left(\frac{P{V}_{a}+{E}_{a}}{{RT}}\right)$$

where ${A}_{k}$ is the pre-exponential constant; $\mu$ is the shear modulus (80 GPa); $d$ is grain size (1 mm); $b$ is the length of the Burgers vector (0.5 nm); $m$ is the grain size exponent.

The viscosity of the lower mantle is determined by bridgmanite^15^, which is the main mineral phase of the lower mantle. The deformation of bridgmanite in our model follows flow laws of Nabarro-Herring creep^48,49^ and pure-climb creep^14,16^, representing the diffusion creep and pure-climb controlled dislocation creep, respectively.

8 $${\dot{\varepsilon }}_{{diff}}=A\frac{\sigma {V}_{m}}{{RT}{d}^{2}}\left({D}^{{lat}}+\frac{\delta {D}^{{gb}}}{d}\right)$$

9 $${\dot{\varepsilon }}_{{dis}}=\frac{{D}^{{lat}}b{\sigma }^{3}}{\pi {k}_{B}T{\mu }^{2}}/{\mathrm{ln}}\left(\frac{4\mu }{\pi \sigma }\right)$$

where $A$ is a constant ($A=3/16$); ${V}_{m}$ is the molar volume (2.55×10^-5^ m^3^ · mol^-1^); $d$ is the grain size (1 mm); $\delta$ is the effective thickness of the grain boundary; $b$ is the Burgers vector (0.5 nm); ${k}_{B}$ is the Boltzmann constant; $\mu$ is the shear modulus (210 GPa); $T$ is temperature; ${D}^{{lat}}$ and ${D}^{{gb}}$ are the lattice and grain-boundary diffusion coefficients, which are calculated according to ref. ^8^:

10 $${D}^{{lat}}=\frac{{D}_{0}^{{lat}}exp \left(-\Delta {H}^{{lat}}+\left(P-25\right)\Delta {V}^{{lat}}\right)}{{RT}}$$

11 $$\delta {D}^{{gb}}=\frac{\delta {D}_{0}^{{gb}}exp \left(-\Delta {H}^{{gb}}+\left(P-25\right)\Delta {V}^{{gb}}\right)}{{RT}}$$

where ${D}_{0}^{{lat}}$ and $\delta {D}_{0}^{{gb}}$ are pre-exponential factors, taking values of 5.10×10^-11^ m^2^·s^-1^ and 7.12 ×10^-17^ m^3^·s^-1^; $\Delta {H}^{{lat}}$ and $\Delta {H}^{{gb}}$ are activation enthalpies at 25 GPa, with values of 3.60×10^5 ^J·mol^-1^ and 3.11×10^5 ^J·mol^-1^; $\Delta {V}^{{lat}}$ and $\Delta {V}^{{gb}}$ are activation volumes, taking values of 2.1×10^-6^ m^3^·mol^-1^ and 4.0 × 10^-6^ m^3^·mol^-1^. The diffusion coefficients ${D}_{0}^{{lat}}$ and $\Delta {H}^{{lat}}$ are those of the slowest diffusion species Si measured by ref. ^49^. Due to a much larger value of ${D}^{{lat}}$ than $\delta {D}^{{gb}}$^8^, the viscosity of the lower mantle is largely determined by ${D}^{{lat}}$. Based on these parameters, we draw the deformation map of the pure climb creep and Nabarro-Herring mechanisms (Supplementary Fig. 3). The pure climb creep dominates the viscous deformation when the stress is high, while the Nabarro-Herring creep dominates when the grain size is small. This is consistent with former studies^16,49^.

The ductile viscosity is decided by the integrated effect of the two creep mechanisms^46^, which is calculated by ${\eta }_{{ductile}}=1/\left(\frac{1}{{\eta }_{{diff}}}+\frac{1}{{\eta }_{{disl}}}\right)$.

Peierls creep dominates the ductile deformation over the dislocation creep^50^ at high stress and low temperature conditions, of which the viscosity is calculated as:

12 $${\eta}_{{peierls}}=\frac{1}{2{A}_{{peierls}}{\sigma}_{{II}}}\exp\left(\frac{{E}_{a}+P{V}_{a}}{{RT}}{\left(1-{\left(\frac{{\sigma}_{{II}}}{{\sigma}_{{peierls}}}\right)}^{p}\right)}^{q}\right)$$

where ${{{\boldsymbol{\sigma }}}}_{{{\boldsymbol{II}}}}$ is the second invariant of stress, ${{{\boldsymbol{\sigma }}}}_{{{\boldsymbol{peierls}}}}$ is the Peierls stress (9.1×10^9 ^Pa), ${A}_{{Peierls}}$, $p$ and $q$ are experimentally derived material constant, with value of 6.3 × 10^-5^Pa^−2^s^-1^, 1, and 2, respectively.

Once reaching the yield stress (${\sigma }_{{II}}{ > =\sigma }_{y}$), plastic deformation takes place. The plastic viscosity is calculated by the Drager-Prager yield criterion:

13 $${\eta }_{{plastic}}=\frac{{\sigma }_{y}}{2{\dot{\varepsilon }}_{{II}}}=\frac{{C}_{0}+P\sin \left({\varphi }_{{dry}}\right)}{2{\dot{\varepsilon }}_{{II}}}$$

where ${C}_{0}$ is the cohesion, ${\varphi }_{{dry}\,}$ is the internal friction coefficient under dry conditions. The plastic strain softening effect is employed; and with the strain accumulating, the ${C}_{0}$ and ${\varphi }_{{dry}\,}$ decrease linearly (Table S1).

The effective viscosity of rocks is determined by the minimum viscosity of different deformation mechanisms:

14 $${\eta }_{{eff}}=min \left({\eta }_{{ductile}},{\eta }_{{peierls}},{\eta }_{{plastic}}\right)$$

### Thermal conductivity

The thermal conductivity is a key parameter controlling the thermal exchange between the cold slab and the ambient hot mantle, which affects the kinetics of phase transformation. In our models, we consider pressure-temperature-composition-dependent (P-T-C) thermal conductivity for oceanic crust and mantle. In the lower mantle, we mainly consider the mineral phase of bridgmanite and stishovite due to a large fraction of bridgmanite and the high thermal conductivity of stishovite^51^. Mineral phases such as ferropericlase, calcium-ferrite oxide (CF), and Fe-NAL phase are not considered due to their lower fractions and similar thermal conductivity of the CF phase and NAL phase to that of bridgmanite^51^. For oceanic crust, the thermal conductivity equals that of eclogite at mantle depth less than 300 km, i.e., *k*_*oc*_=*k*_*ecl*_. When stishovite is present at a depth of over 300 km, the thermal conductivity of the oceanic crust is calculated by *k*_*oc*_ = 0.9*k*_*ecl*_ + 0.1*k*_*stv*_, assuming a 10% fraction of stishovite. At the lower mantle, considering the delayed post-garnet transformation, the thermal conductivity of oceanic crust is obtained by a linear combination of bridgmanite, eclogite, and stishovite, i.e., *k*_*oc*_ = *F*×*k*_*brg*_ + (1-0.2-*F*×*k*_*ecl*_ + 0.2*k*_*stv*_, *F* is the fraction of post-garnet phases. For mantle rocks, the thermal conductivity of the upper mantle follows that of ref. ^52^, while the thermal conductivity of the lower mantle follows that of ref. ^53^.

15 $${k}_{{ecl}}=\left(1.94+\frac{372}{T}+\frac{29198}{{T}^{2}}\right)\times \left(1+0.038P\right)$$

16 $${k}_{{stv}}=	({4.7497 \times {10}^{-6}P}^{4}-5.8777\times {10}^{-4}{P}^{3}+2.61{\times {10}^{-2}P}^{2} \\ 	-0.70667{P}^{1}+75.70)\times {\left(\frac{T}{298}\right)}^{-1}$$

17 $${k}_{{brg}}=\left(4.9+0.105P\right)f\left(\frac{T}{1200}\right)\left(\frac{1200}{T}\right)$$

where ${k}_{{ecl}}$ is the thermal conductivity of eclogite which is derived from ref. ^54^; ${k}_{{stv}}$ is the thermal conductivity of stishovite which is according to ref. ^51^; ${k}_{{brg}}$ is the thermal conductivity of bridgmanite; $P$ is the pressure in GPa; $T$ is the temperature in Kelvin; $f$ is a function^53^.

### Model setup

#### Composition and boundary conditions

The physical dimension of our model is 10,000 × 2800 km (Supplementary Fig. 4), discretized with a non-uniform grid with 1001 × 701 nodes. The grid resolution is refined at the subduction zone to 4 × 2 km (x and y directions). A simple subduction system is applied in our model (Supplementary Fig. 4). The upper and lower plates are separated by an inclined weak zone with a 25 ° dip angle. The upper plate is composed of three layers, including the upper continental crust (20 km), lower continental crust (10 km), and lithospheric mantle (95 km). The lower plate is a typical oceanic plate consisting of a 2-km-thick layer of altered basalts, a 5-km-thick layer of gabbroic rocks, and a lithospheric mantle (93 km). The oceanic lithospheric mantle comprises two layers, including a 53-km-thick depleted layer (harzburgite) and a 40-km-thick less depleted layer (lherzolite) according to ref. ^55^. The harzburgite layer is applied with a lower initial density (3.25 g⋅cm^-3^) while the lherzolite has the same initial density as the upper mantle. Check Table S1 for the parameters of rock rheology and physical properties. A linear temperature structure is applied for the continental lithosphere, with a Moho temperature of 600 °C and lithosphere-asthenosphere boundary temperature of 1360 °C. The thermal structure of the oceanic plate follows the plate-cooling model^56^ with a plate bottom temperature of 1360 °C, plate age of 80 Myr, and thermal diffusivity of 1 mm^2^ ⋅ s^-1^. The sublithospheric mantle follows an adiabatic gradient of 0.4 °C⋅km^−1^.

Free slip is applied on all boundaries. Two internal pushing velocities at 1500 km (with 4 cm⋅yr^-1^) and 8000 km (with 1 cm⋅yr^-1^) are prescribed to initiate subduction (Supplementary Fig. 4). The pushing velocities last 16 Myr until the slab reaches the lower mantle. We then withdraw the two velocities and leave subduction driven by the slab’s buoyancy. The thermal boundary conditions are set as follows: 0 °C at the top boundary, a constant value of 2,370 °C at the lower boundary, and thermal insulation conditions at both side boundaries with no heat flux across.

#### Metastable garnet content in the slab

The sluggish kinetics of post-garnet transformation enable the preservation of metastable garnet in subducted slabs within the uppermost lower mantle. High-pressure experiments suggest that the relevant metastable end-members are Al-rich garnets such as pyrope, grossular, and almandine, rather than majorite, as the latter transforms to bridgmanite more rapidly^22^. Although these garnet end-members become progressively majoritic above mantle pressures above ~5–6 GPa through pyroxene dissolution^57^, the kinetics of this conversion are sluggish at low slab temperatures^17^. This allows the original garnet assemblages to survive largely unaltered into the lower mantle. Therefore, we take the upper-mantle (<6 GPa) garnet content as the initial inventory of metastable garnet in our models, assuming minimal majoritic conversion. Due to the current lack of growth kinetics data for grossular and almandine and given their similar decomposition mechanisms to pyrope^36^, we apply the same growth kinetics to all garnet end-members in this study.

For basaltic and gabbroic crust, high-pressure experiments revealed a garnet content of ~20–35% at the upper mantle conditions^43,57,58^, while the thermodynamic calculation suggested a garnet content of 40–60%^59,60^(Supplementary Fig. 5). For harzburgitic mantle, high-pressure experiments and our calculation (Supplementary Fig. 5) revealed a garnet content of less than 10% at the upper mantle conditions^61,62^, while some of the natural samples and thermodynamic calculations suggested the garnet content may exceed 10%^44,59^. For lherzolitic and pyrolitic mantle, high-pressure experiments and thermodynamic calculations suggest a range of 10–20% for garnet content at the upper mantle conditions^59,61,63^ (Supplementary Fig. 5). The different garnet content in former studies for slab components is mainly induced by the different chemical composition and temperature conditions used in the experiment and calculation. Based on these, we consider a range of initial contents of metastable garnet for each slab component in our models: 25–50% in basaltic and gabbroic crust, 0–10% in harzburgitic mantle, and 10–15% in lherzolitic mantle.

## Supplementary information

Supplementary information

Transparent Peer Review file

## Data Availability

The relevant data and model outputs presented in this study are available at https://figshare.com/articles/dataset/Shen_Yang_Zhao_NC2025/30443903. Correspondence and requests for materials should be addressed to Jianfeng Yang.

## Code availability

The numerical code I2VIS was originally developed by Taras Gerya (taras.gerya@erdw.ethz.ch) and slightly modified by M. Faccenda and J. Yang. Requests for the code can contact the main code developer or the corresponding author. Figures are produced by the open-source code Generic Mapping Tools (https://www.generic-mapping-tools.org, Version 6.5.0).
