# Supplementary material for: Sluggish post-garnet transformation controls slab stagnation at the uppermost lower mantle
Source: Nat Commun. 2026 Apr 23;17:5647. doi: 10.1038/s41467-026-72495-5 (PMC13315703; doi:10.1038/s41467-026-72495-5)
Supplement: Supplementary file 1 — Supplementary information [file 41467_2026_72495_MOESM1_ESM.pdf]

Supplementary information

Sluggish post-garnet transformation controls slab stagnation at the uppermost lower mantle

Yongqiang Shen[1], Jianfeng Yang [1, 2*], Liang Zhao[3,2]

[1]State Key Laboratory of Lithospheric and Environmental Coevolution, Institute of Geology and Geophysics, Chinese Academy of Sciences, Beijing, China

[2]College of Earth and Planetary Sciences, University of Chinese Academy of Sciences, Beijing, China

[3]Key Laboratory of Deep Petroleum Intelligent Exploration and Development, Institute of Geology and Geophysics, Chinese Academy of Sciences, Beijing, China

Supplementary Figures

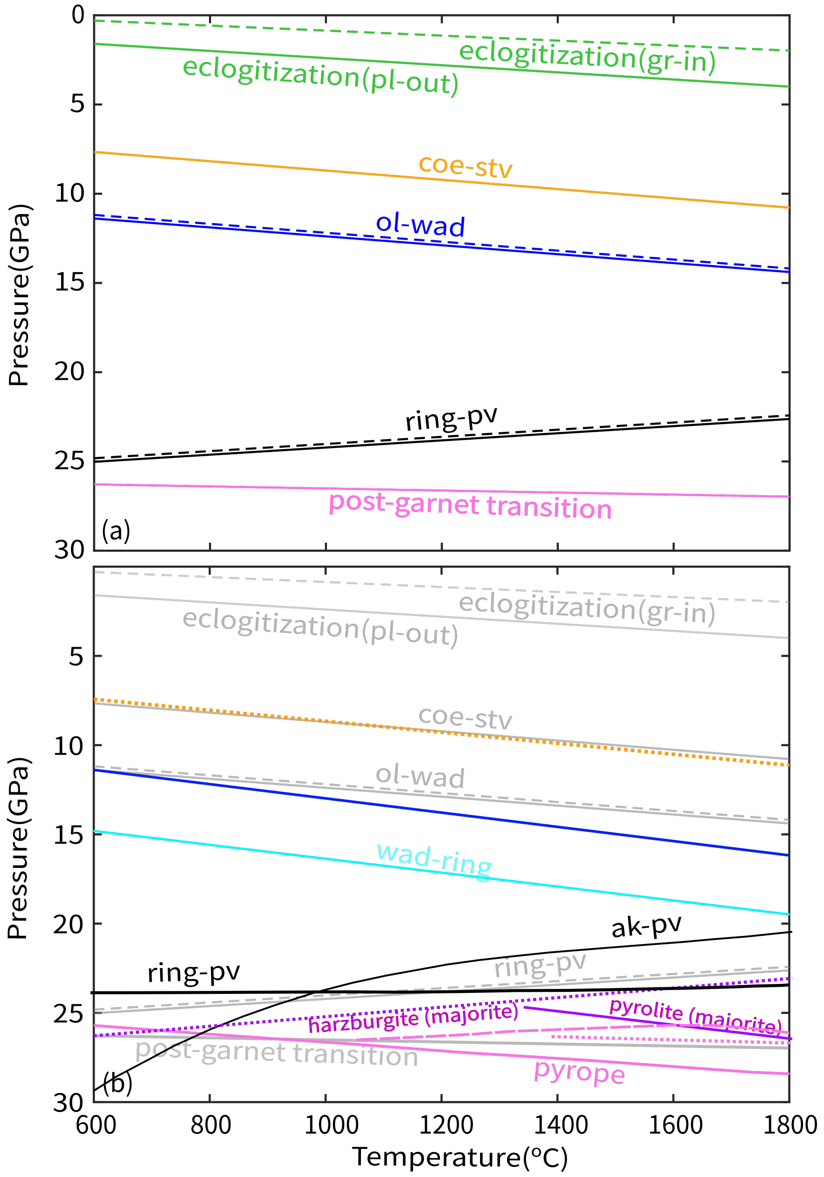

Fig. S1. Phase transition boundaries. a, Phase boundaries considered in our numerical models. Phase boundaries of eclogite transformation are based on experimental data of Ito and Kennedy[1]. The coesite-stishovite (coe-stv) transition is according to the experimental results of Zhang et al.[2]. The phase boundaries of the olivine-wadsleyite (ol-wad) transition and ringwoodite-perovskite (ring-pv) transition are derived from Katsura and Ito[3] and Ito et al.[4], respectively. Phase boundary of post-garnet transition is based on experiments on the composition of basaltic crust derived from Hirose et al.[5]. **b**, Alternative phase boundaries from other studies for comparison and sensitivity tests. The grep lines correspond to phase boundaries shown in Fig. S1a. Other phase boundaries and corresponding references are: coe-stv (grey dotted line)-Ono et al. (2017)[6], ol-wad (dark blue line)-

Morishima et al. (1994)[7], wad-ring (light blue line)-Katsura and Ito (1989)[3], ak-pv (thin black line)-Chanyshev et al. (2022)[8], ring-pv (thick black line)-Chanyshev et al. (2022)[8], post-garnet transition of majorite in pyrolitic mantle (purple line)-Ishii et al. (2011)[9], post-garnet transition of majorite in harzburgitic mantle (purple dashed line)-Ishii et al. (2019)[10], post-garnet transition of pyrope (pink long dashed line)-Ishii et al. (2023)[11], post-garnet transition of pyrope (pink dotted line) -Kubo and Akaogi (2000)[12], post-garnet transition of pyrope (pink solid line)-Wang and Wu (2017)[13].

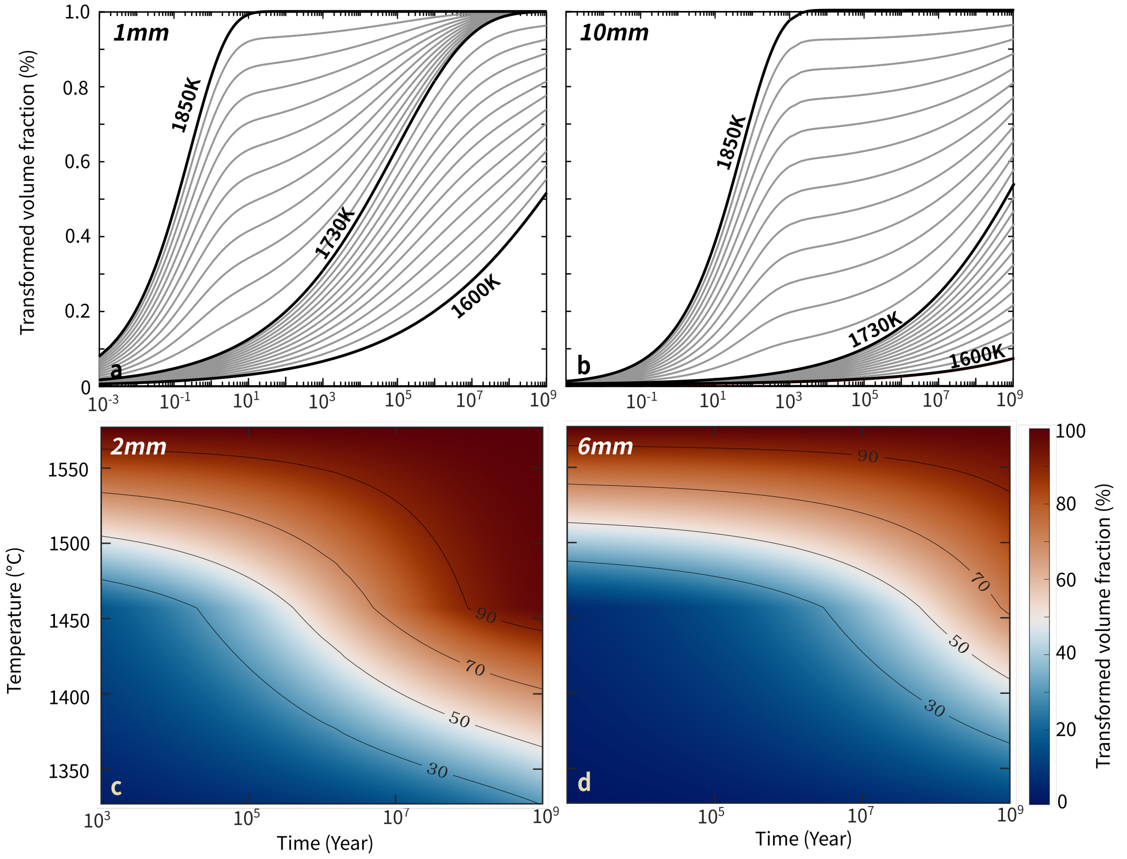

Fig. S2. Degree of post-garnet transformation versus time. The transformed volume fraction of garnet over time for the grain size of **a,** 1 mm, and **b,** 10 mm, at different temperatures. The black curves labeled by 1600 K, 1730 K, and 1850 K are obtained using the transformation rate equation (A4) and kinetic parameters determined by Kubo et al.[14]. The grey curves are the results of interpolation based on the three black curves. **c**, **d**, Distribution of transformed garnet fraction with temperature and growth time of post-garnet phase for grain sizes of 2 mm and 6 mm, respectively.

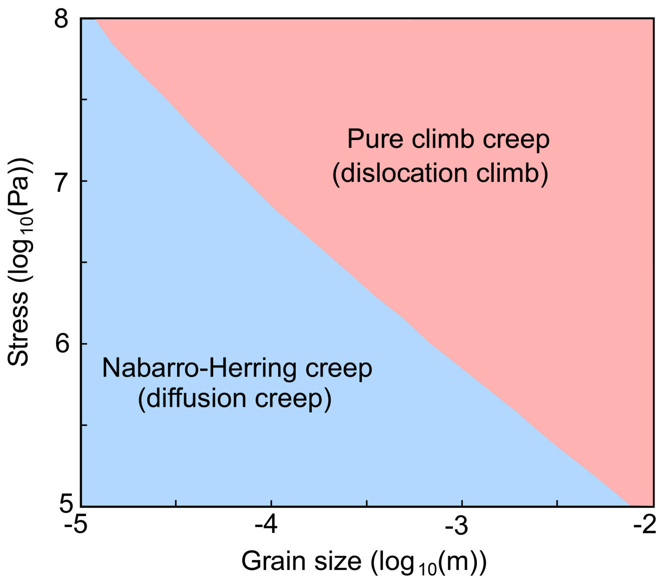

Fig. S3. Deformation mechanism map at 35 GPa, 2,000 K, comparing pure climb creep and Nabarro-Herring mechanisms. See the rock rheology section for the formula used in the calculation and the selection of parameters.

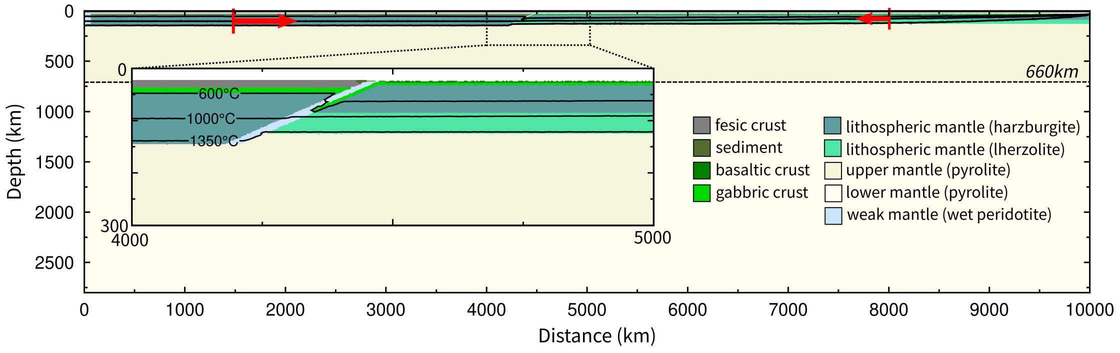

Fig. S4. Initial model setup. A simple subduction system composed of the upper plate (left) and lower plate (right) is designed. Two red arrows indicate the pushing velocities of 4 cm/yr and 1 cm/yr on the upper and lower plates, respectively.

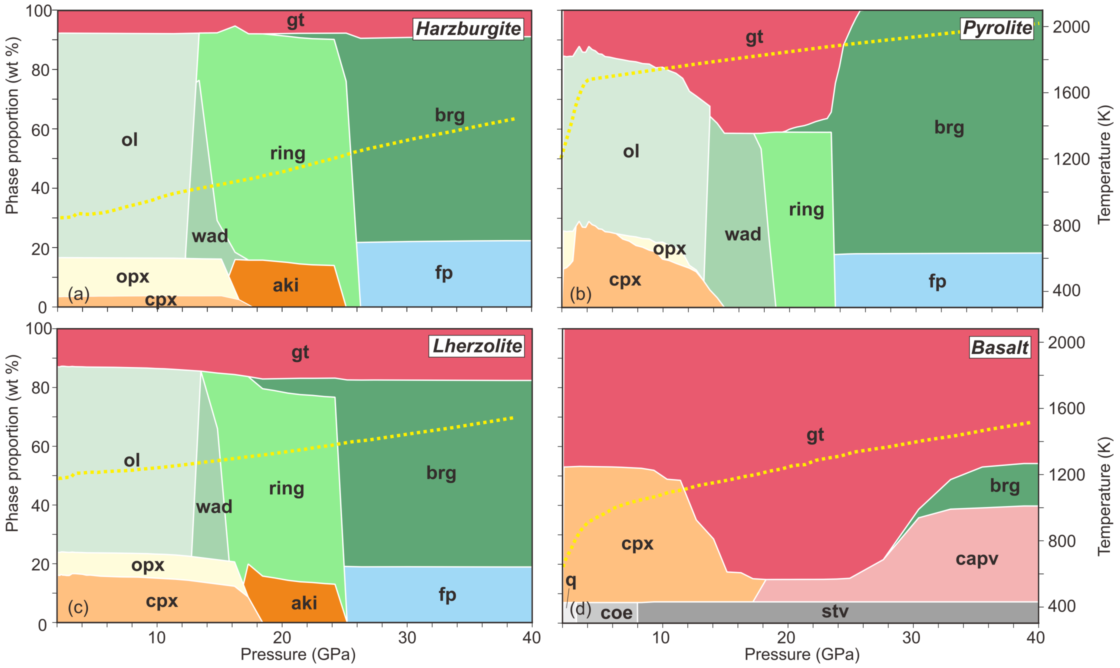

Fig. S5. Mineral phase proportions for different chemical compositions across the uppermost lower mantle pressure range. a, Harzburgitic mantle. **b**, Pyrolitic mantle. **c**, Lherzolitic mantle. **d**, Basaltic crust. Phase proportions were computed using the thermodynamic software Perple_X[15]. Non-equilibrium conditions arising from sluggish post-garnet transformation are accounted for in phase-diagram calculations of harzburgite, lherzolite, and basalt because these phases are in low-temperature states during slab subduction. To approximate non-equilibrium conditions, aluminum-bearing bridgmanite, calcium-ferrite phase, new aluminous phase, and corundum were excluded from the calculations. This allows aluminum-rich garnet (e.g., pyrope) to persist without decomposition to depths in the uppermost lower mantle. Yellow dashed lines indicate representative pressure–temperature paths for each slab component, derived from the reference model. Check Tables S2 and S3 for the chemical compositions of each slab component and the selection of solution models used in the calculation. Abbreviations: q-quartz, coe-coesite, stv-stishovite, gr-garnet, cpx-clinopyroxene, opx-orthopyroxene, ol-olivine, brg-bridgmanite, fp-ferropericlase, capv-calcium silicate perovskite, wad-wadsleyite, ring-ringwoodite, aki-akimotoite.

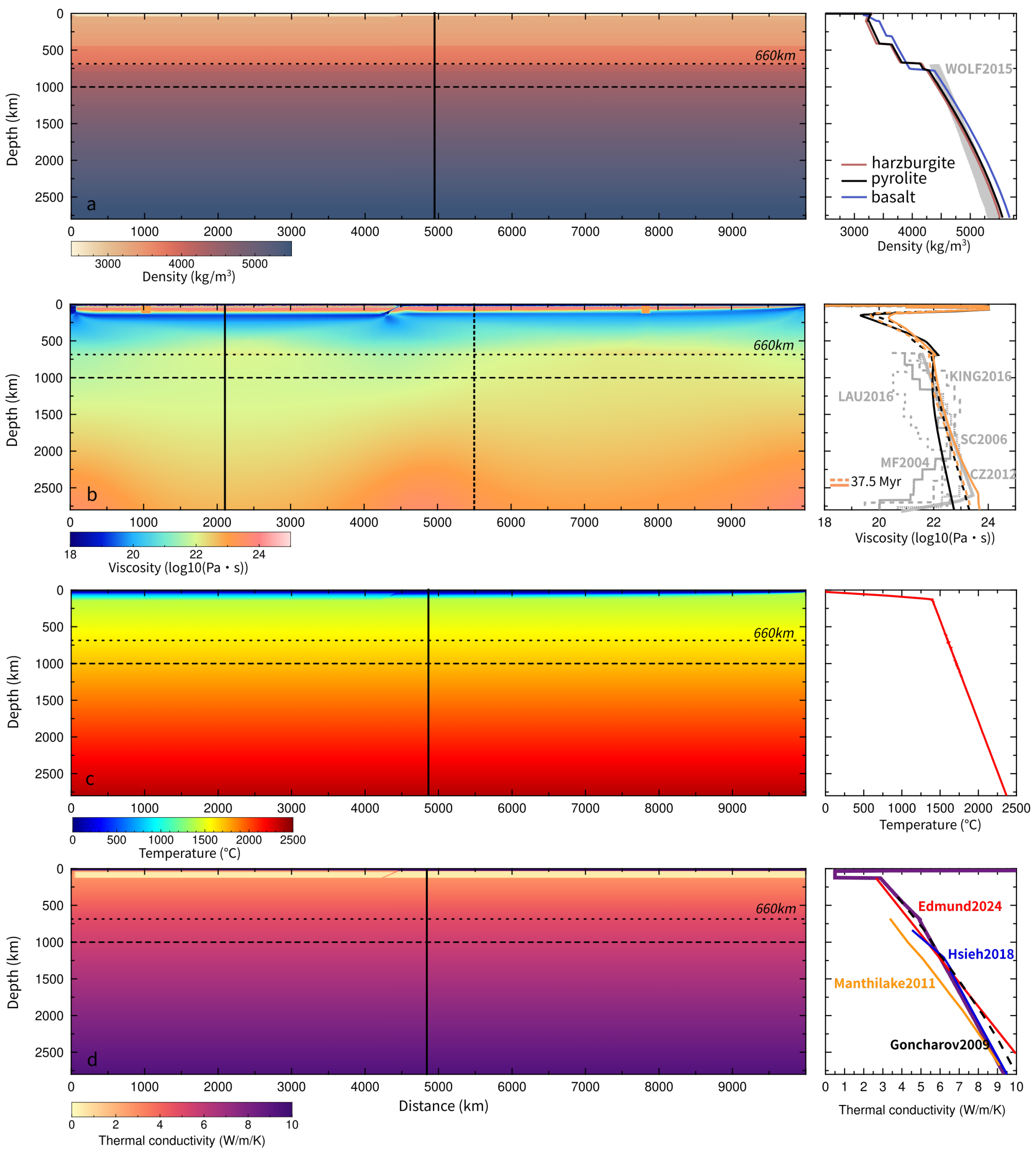

Fig. S6. Model setup for physical properties. **a**, The initial density distribution and a vertical profile on the left and right panels, respectively. The color-coded lines are density profiles of different compositions at our initial model conditions. The grey area denotes the density range of the lower mantle proposed by Wolf et al.[16], which accounts for the variable Fe content of bridgmanite. The black line denotes the position where the density profiles are calculated. **b,** The initial viscosity distribution and two vertical profiles on the left and right panels, respectively. The viscosity field at 0.5 Myr is shown here. Profiles at two moments are present: (1) the profiles at 0.5 Myr, denoted by black color; (2) profiles at 37.5 Myr (see Fig. 3 in the main text), denoted by orange color. The viscosity profiles are further compared with former studies: LAU2016-Lau et al. (2016)[17], KING2016-King et al. (2016)[18], SC2006-Steinberger

and Calderwood (2006)[19], MF2004-Mitrovica and Forte (2004)[20], CZ2012-Cizkova et al. (2012)[21]. **c**, Temperature distribution and profile. **d**, Distribution of mantle thermal conductivity and profile. The thermal conductivity profile is compared with previous studies, including Edmund et al. (2024)[22], Hsieh et al. (2018)[23], Manthilake et al. (2011)[24], and Goncharov et al. (2009)[25]. The thermal conductivity of oceanic lithospheric mantle at depths shallower than 120 km is set to be 0.5 $W \cdot m^{-1} \cdot K^{-1}$ to keep a steady thermal state of the oceanic plate.

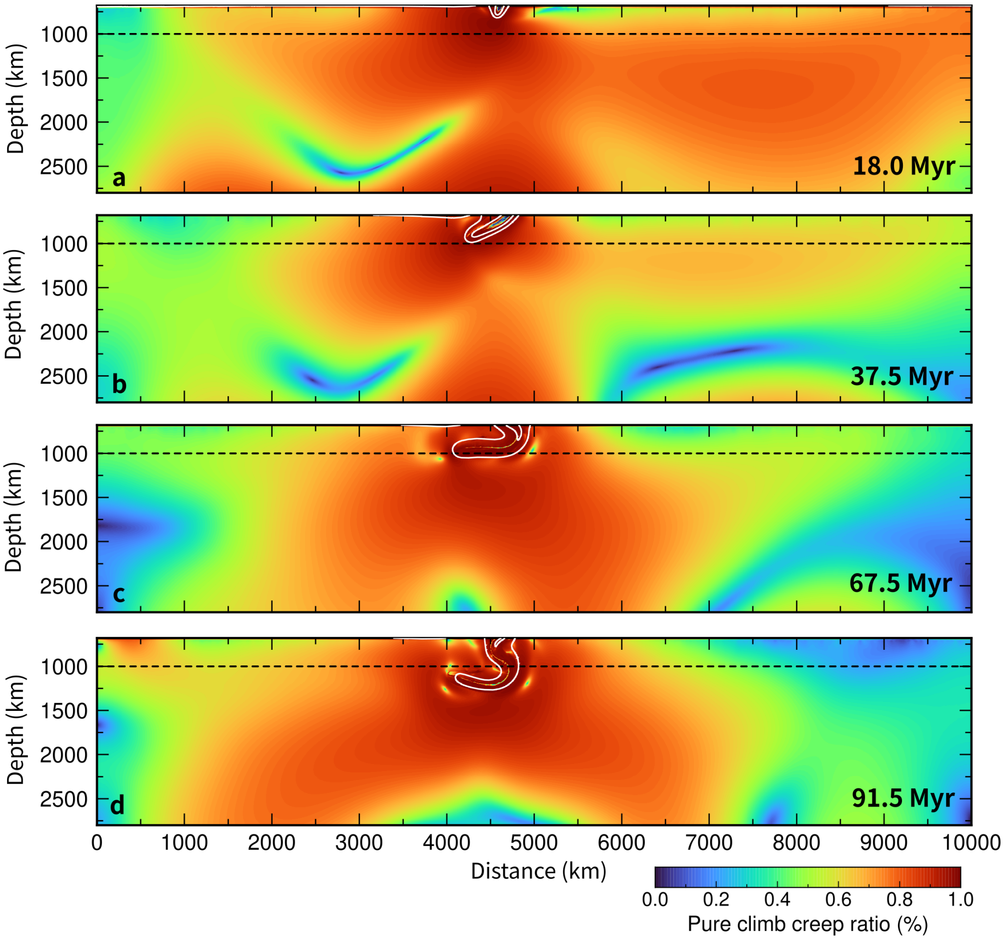

Fig. S7. Pure climb creep ratio of lower mantle in the reference model. The pure climb creep ratio is determined by its contribution to the mantle viscosity, i.e., the ratio of pure climb creep viscosity (η_{disl}) to the total viscosity, which is a combination of both diffusion creep viscosity (η_{diff}) and dislocation creep viscosity (η_{disl}). The white lines are isothermal contours of 1,300°C and 1,600°C.

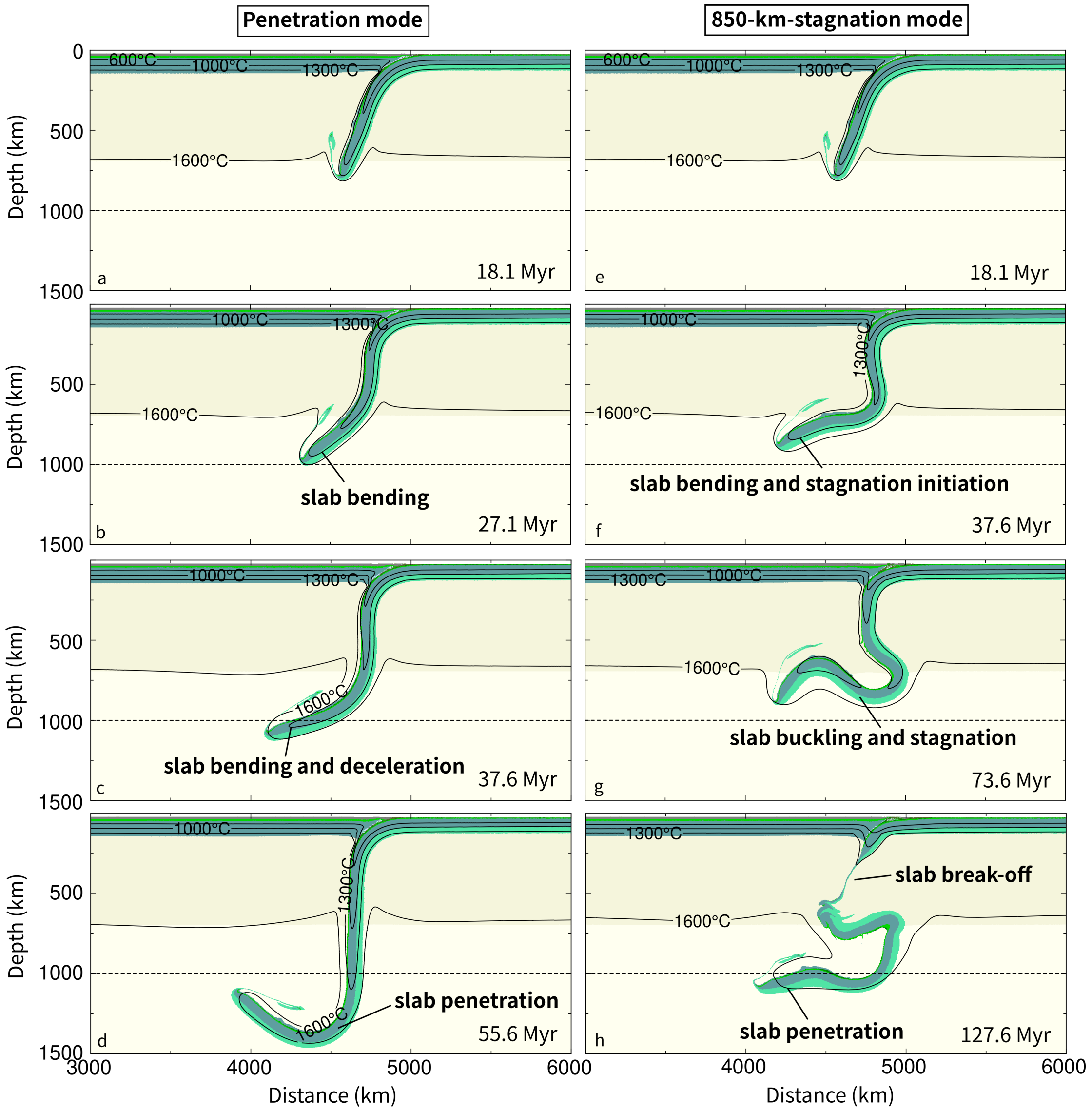

Fig. S8. Typical model evolution results in the penetration regime and 850-km-stagnation regime. The initial contents of metastable garnet in the penetration model are: oceanic crust-30%, harzburgitic lithosphere-4%, and lherzolitic lithosphere-15%. The initial contents of metastable garnet in the 850-km-stagnation model are: oceanic crust-50%, harzburgitic lithosphere-8%, lherzolitic lithosphere-15%. The garnet grain size in the penetration mode is prescribed as 4mm, while that in the 850-km-stagnation model is set to be 2 mm.

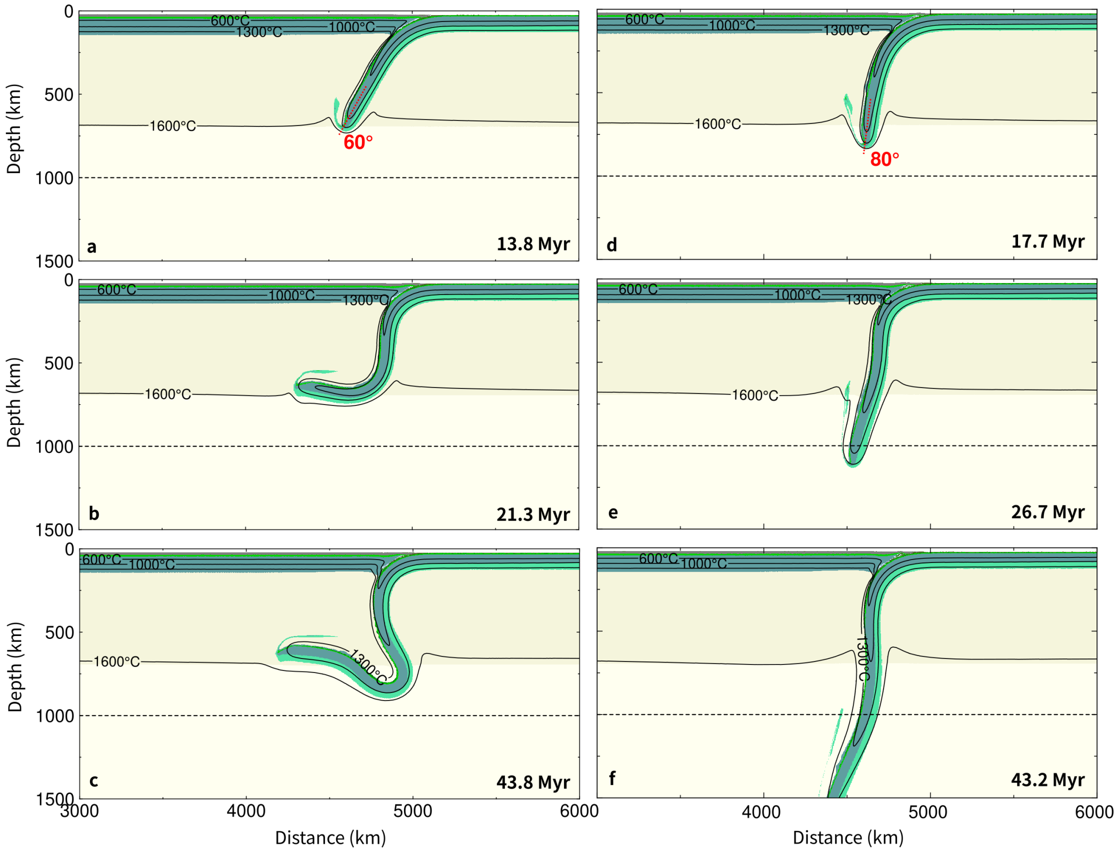

Fig. S9. Model results with different slab geometry. The slab geometry refers to the dip angle of the slab at the interface of the upper and lower mantle. **a-c**, Slab dip angle of 60°. **d-f**, Slab dip angle of 80°. The change in dip angle is realized by varying the duration of the push velocity. The duration of push velocity for the two cases is 17 Myr and 15 Myr, respectively.

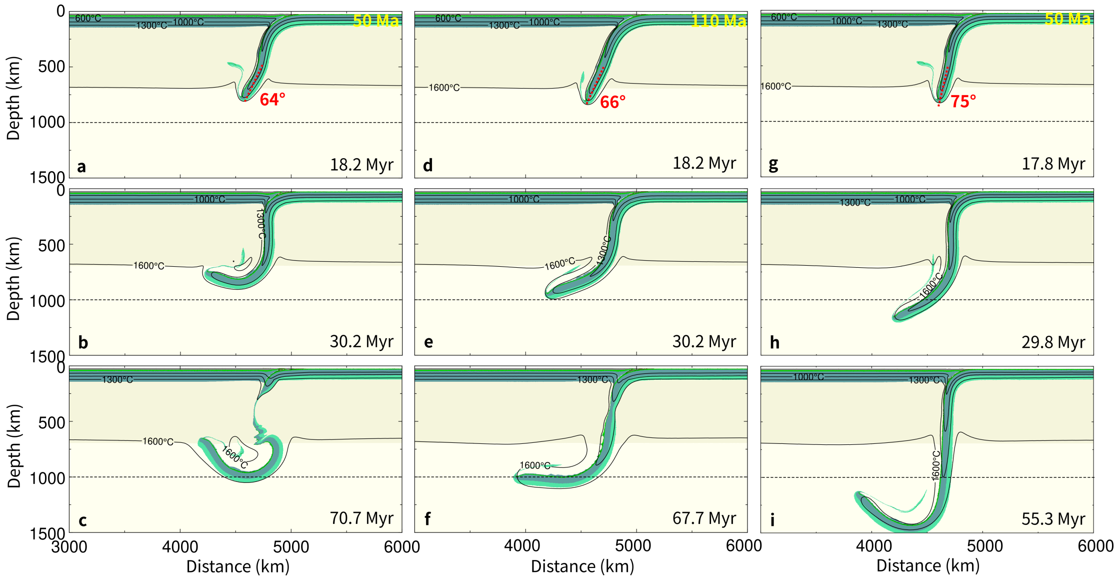

Fig. S10. Model results with different slab ages. a-c, Slab age of 50 Ma. **d-f**, Slab age of 110 Ma. **g-i**, Slab age of 50 Ma with high dip angle (75°). The dip angle is achieved by applying a

push velocity for 15.5 Myr.

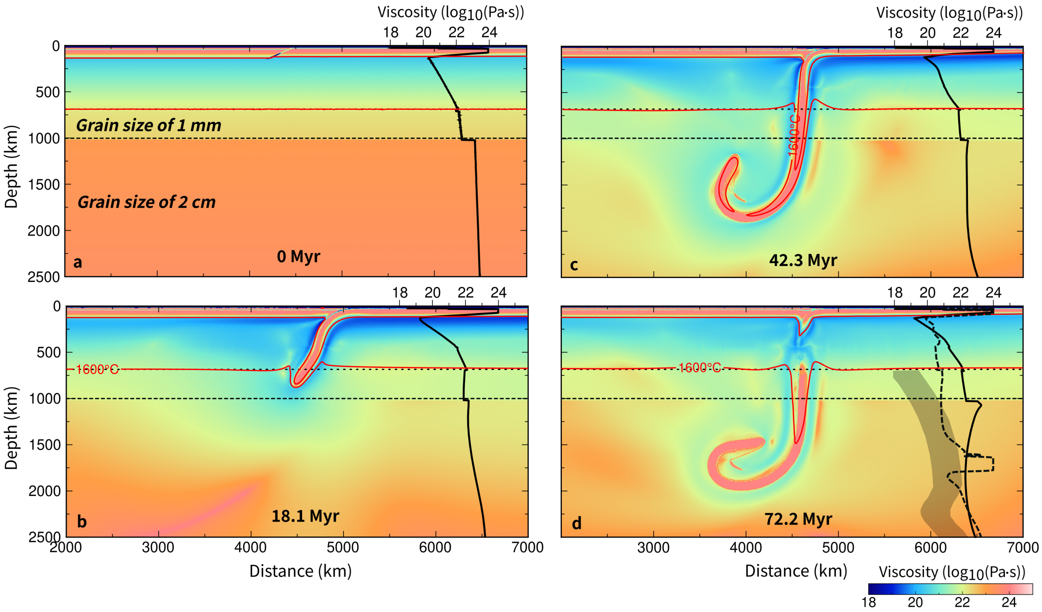

Fig. S11. Model results with lower mantle viscosity increase due to larger grain sizes. The viscosity jump is realized by increasing the grain size of bridgmanite at depths larger than 1000 km to 2 cm according to Fei et al.[26]. The strain rate of the whole mantle at 0 Myr is prescribed as 10^{-18} s^{-1}, leading to a viscosity increase of ~1 order of magnitude at 1000 km depth. The solid black lines show the viscosity profiles at Distance = 6500 km. The red lines represent isothermal contours of 1300°C and 1600°C. The vertical dashed line in **d** denotes the viscosity profile at a distance of 4450 km, which is across the mantle area surrounding the slab with a high strain rate. The grey area in **d** denotes the viscosity range, accounting for the ferropericlase strengthening proposed by Marquardt and Miyagi[27].

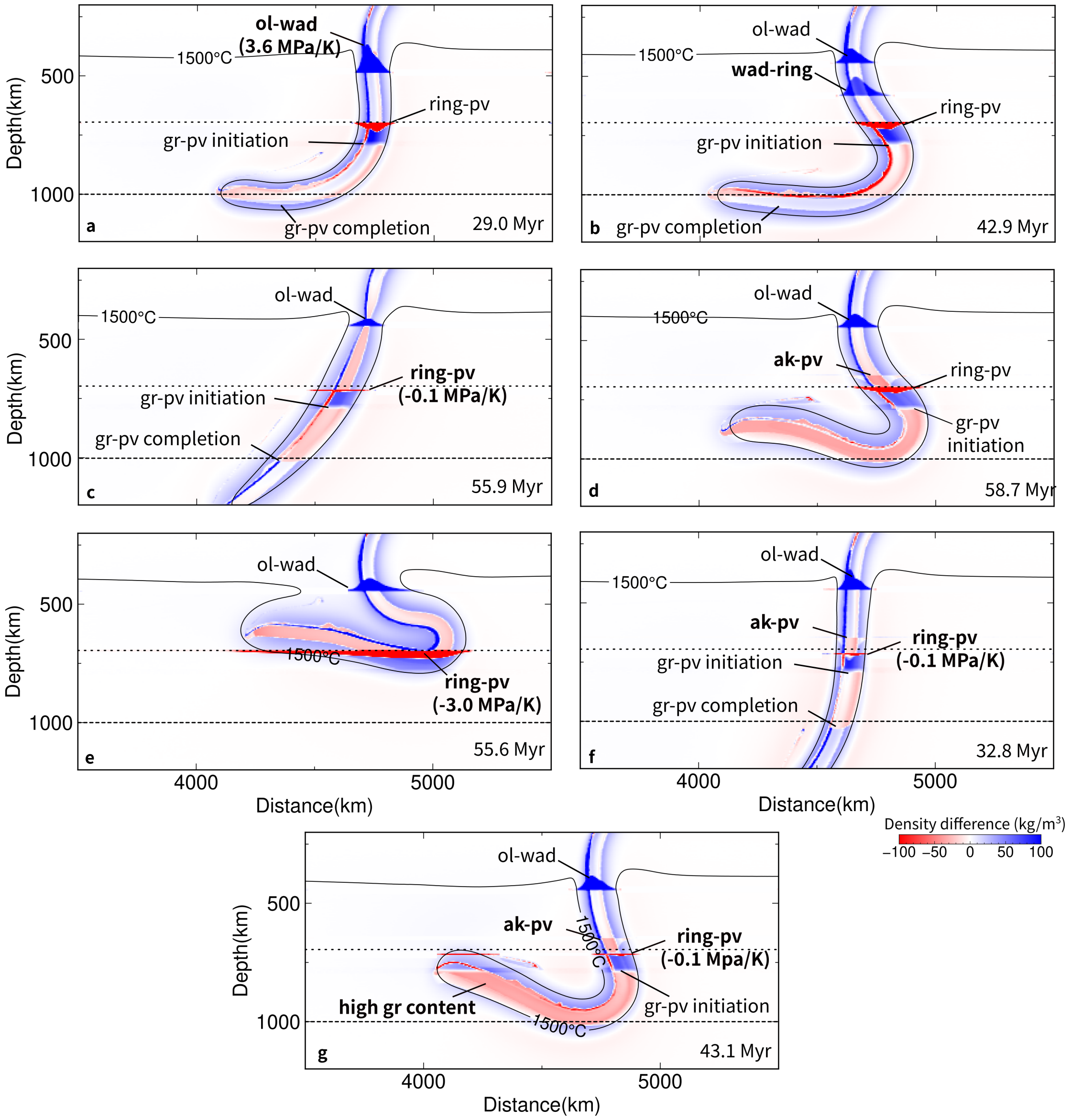

Fig. S12. Model tests on phase transitions in the mantle transition zone. The model evolution is illustrated by density differences relative to the density profile averaged from a distance of 0 to 2000 km. The two dashed lines denote 660-km depth and 1000-km depth, respectively. **a**, The olivine (ol)-wadsleyite (wad) transition with a steep Clapeyron slope (3.6 MPa/K). The phase boundary is derived from Morishima et al. (1994)[7]. **b**, The wadsleyite-ringwoodite (ring) transition. The phase boundary is derived from Katsura and Ito (1989)[3]. **c**, The ringwoodite-perovskite transition with a shallow Clapeyron slope (-0.1 MPa/K) based on the study of Chanyshev et al. (2022)[8]. **d**, Model test on akimotoite (ak)-perovskite (pv) transition based on the study of Chanyshev et al. (2022)[8]. The akimotoite content in harzburgitic mantle, lherzolitic mantle, and pyrolitic mantle is set up to be 20%, 10%, and 10%, respectively, based on Ishii et al. (2019)[10] and Hirose (2002)[28]. **e**, The ringwoodite-perovskite transition with

a steep Clapeyron slope (-3.0 MPa/K). **f**, The integrated effect of ring-pv and ak-pv transitions based on the study of Chanyshev et al. (2022)[8]. **g**, Model results based on that shown in Fig. S12f with additional higher garnet content (8% for harzburgite, 45% for basalt) and smaller slab dip angle (65°).

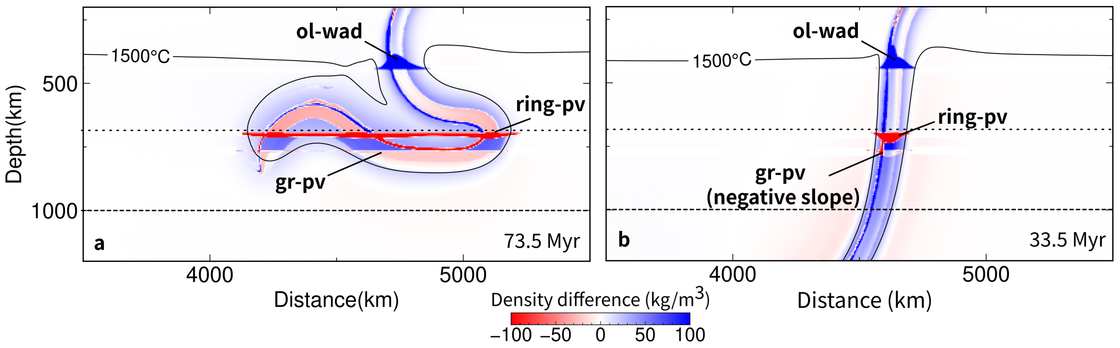

Fig. S13. Model results with a negative Clapeyron slope of the post-garnet transition. The model evolution is illustrated by density differences relative to the density profile averaged over the range 0–2000 km. The two dashed lines denote depths of 660 km and 1000 km, respectively. **a,** Model results based on the reference model. **b,** Model results with rapid post-garnet transition. Abbreviations: olivine (ol), wadsleyite (wad), ringwoodite (ring), perovskite (pv), garnet (gr).

Table S1. Physical parameters

	Sediments	Upper continental crust	Lower continental crust	Oceanic crust	Upper mantle	Lower mantle	Weak zone	Reference [6]
Flow Laws	Wet quartzite	Felsic Granulite	Plagioclase (An75)	Plagioclase (An75)	Dry olivine	Bridgmanite	Wet olivine	
ρ (kg/m^3)	2650	2750	3000	3000	3300	3300	3300	1
φ_0	0.0	0.15	0.6	0.3	0.6	0.6	0.0	2
φ_1	0.0	0.15	0.2	0.15	0.2	0.2	0.0	2
E_a (J/mol)	1.54×10^5	2.43×10^5	1.54×10^5	2.38×10^5	KW1993[4]	PCC/NH[5]	4.7×10^5	2
V_a (J/MPa/mol)	8	8	8	8	KW1993	PCC/NH	8	2
A_D (Pa^n·s)	1.97×10^{17}	4.97×10^{20}	1.97×10^{17}	4.80×10^{22}	KW1993	PCC/NH	5.01×10^{20}	2
n	2.3	3.1	2.3	3.2	KW1993	PCC/NH	4.0	2
H_r (μW/m^3)	2.0	2.0	0.22	0.24	0.022	0.022	0.022	1
k (W/m/K)	$[0.64+807/(T+77)] \times \exp(0.04\ P_{GPa})$	$[0.64+807/(T+77)] \times \exp(0.04P_{GPa})$	$[1.18+470/(T+77)] \times \exp(0.04P_{GPa})$	P-T-C dependent[3]	P-T-C dependent	P-T-C dependent	P-T-C dependent	3
α (1/K) [1]	2×10^{-5}	2×10^{-5}	2.5×10^{-5}	2.5×10^{-5}	2.5×10^{-5}	2.5×10^{-5}	2.5×10^{-5}	1
β (1/GPa) [2]	4.5×10^{-3}	4.5×10^{-3}	4.5×10^{-3}	6.0×10^{-3}	6.0×10^{-3}	6.0×10^{-3}	6.0×10^{-3}	1

Note: [1] The thermal expansion coefficient of upper mantle rocks is constant but linearly decreases in the lower mantle to a value of 1.0×10^{-5} K^{-1} at the bottom of the lower mantle.

[2] The compressibility of upper mantle rocks is constant, but it decreases linearly in the lower mantle to 4.0×10^{-3} GPa^{-1} at the bottom of the lower mantle.

[3] See the thermal conductivity section for a detailed explanation.

[4] The flow law of Karato and Wu (1993)[29] is applied to the upper mantle in this study. The activation volume (V_a) of dry dislocation creep is 1.0 J/MPa/mol in this study.

[5] PCC-pure climb creep, NH-Nabarro-Herring creep. Check the rock rheology section for lower-mantle viscosity calculation and selection of rheological parameters.

[6] 1–Turcotte and Schubert (2014)[30], 2–Ranalli (1995)[31], 3–Clauser and Huenges (1995)[32]

Table S2. Model rock composition (wt%)

	SiO_2	Al_2O_3	FeO	MgO	CaO	Na_2O	K_2O	References
Basalt	51.75	15.07	10.69	7.77	11.68	2.86	0.16	Gale et al. (2003)[33]
Harzburgite	43.92	1.87	8.17	44.80	1.21	0.02	0.00	Laporte et al. (2004)[34]
Lherzolite	45.00	3.63	8.20	39.68	3.48	0.30	0.00	Hirose and Kushiro (1993)[35]
Pyrolite	45.26	4.03	8.28	39.21	3.21	0.13	0.00	Workman and Hart (2005)[36]

Table S3. Phases and thermodynamic data sources[1]

**Phase**	**Symbol**	**Note/source**
Garnet	Gt(H)	Holland et al. (2013)[37]
Olivine	O(JH)	Jennings and Holland (2015)[38]
Clinopyroxene	Cpx(JH)	Jennings and Holland (2015)
Orthopyroxene	Opx(JH)	Jennings and Holland (2015)
HP_clinopyroxene	Hpx(H)	Holland et al. (2013)[37]
Spinel	Sp(HP)	Holland and Powell (1998)[39]
Binary-feldspar	Pl(h)	Newton et al. (1981)[40]
Bridgmanite	Mpv(H)	Holland et al. (2013)[37]
Calcium silicate perovskite	Cpv(H)	Holland et al. (2013)[37]
Ferropericalse	Fper(H)	Holland et al. (2013)[37]
Wadsleyite	Wad(H)	Holland et al. (2013)[37]
Ringwoodite	Ring(H)	Holland et al. (2013)[37]
Akimotoite	Aki(H)	Holland et al. (2013)[37]

The internally consistent thermodynamic data of Holland et al. (2018)[41] are used to calculate the pseudosection.

References

1. Ito, K. & Kennedy, G. C. An Experimental Study of the Basalt-Garnet Granulite-Eclogite Transition. in *Geophysical Monograph Series* (ed. Heacock, J. G.) 303–314 (American Geophysical Union, Washington D. C., 2013). doi:10.1029/GM014p0303.
2. Zhang, J., Li, B., Utsumi, W. & Liebermann, RobertC. In situ X-ray observations of the coesite-stishovite transition: reversed phase boundary and kinetics. *Phys Chem Minerals* **23**, (1996).
3. Katsura, T. & Ito, E. The system Mg_2 SiO_4 -Fe_2 SiO_4 at high pressures and temperatures: Precise determination of stabilities of olivine, modified spinel, and spinel. *J. Geophys. Res.* **94**, 15663–15670 (1989).
4. Ito, E., Akaogi, M., ToPoR, L. & Navrotsky, A. Negative Pressure-Temperature Slopes for Reactions Foriing MgSiO3 Perovskite from Calorimetry. *Science* **249**, 1275–1278 (1990).
5. Hirose, K., Fei, Y., Ma, Y. & Mao, H.-K. The fate of subducted basaltic crust in the Earth's lower mantle. *Nature* **397**, 53–56 (1999).
6. Ono, S., Kikegawa, T., Higo, Y. & Tange, Y. Precise determination of the phase boundary between coesite and stishovite in SiO 2. *Physics of the Earth and Planetary Interiors* **264**, 1–6 (2017).
7. Morishima, H. *et al.* The Phase Boundary Between a- and P-Mg2SiO4 Determined by in Situ X-ray Observation. *Science* **265**, 1202–1203 (1994).
8. Chanyshev, A. *et al.* Depressed 660-km discontinuity caused by akimotoite–bridgmanite transition. *Nature* **601**, 69–73 (2022).

9. Ishii, T., Kojitani, H. & Akaogi, M. Post-spinel transitions in pyrolite and Mg2SiO4 and akimotoite–perovskite transition in MgSiO3: Precise comparison by high-pressure high-temperature experiments with multi-sample cell technique. *Earth and Planetary Science Letters* **309**, 185–197 (2011).
10. Ishii, T., Kojitani, H. & Akaogi, M. Phase Relations of Harzburgite and MORB up to the Uppermost Lower Mantle Conditions: Precise Comparison With Pyrolite by Multisample Cell High-Pressure Experiments With Implication to Dynamics of Subducted Slabs. *JGR Solid Earth* **124**, 3491–3507 (2019).
11. Ishii, T. *et al.* Buoyancy of slabs and plumes enhanced by curved post-garnet phase boundary. *Nat. Geosci.* **16**, 828–832 (2023).
12. Kubo, A. & Akaogi, M. up to 28 GPa: phase relations of garnet, ilmenite and perovskite.
13. Wang, D., Wu, Z. & Deng, X. Thermal Conductivity of Hydrous Wadsleyite Determined by Non-Equilibrium Molecular Dynamics Based on Machine Learning. *Geophysical Research Letters* **49**, e2022GL100337 (2022).
14. Kubo, T. *et al.* Kinetics of the post-garnet transformation: Implications for density and rheology of subducting slabs. *Physics of the Earth and Planetary Interiors* **170**, 181–192 (2008).
15. Connolly, J. A. D. Computation of phase equilibria by linear programming: A tool for geodynamic modeling and its application to subduction zone decarbonation. *Earth and Planetary Science Letters* **236**, 524–541 (2005).
16. Wolf, A. S., Dera, P. & Prakapenka, V. B. The thermal equation of state of (Mg, Fe)SiO_3 bridgmanite (perovskite) and implications for lower mantle structures. *JGR Solid Earth*

120, 7460–7489 (2015).

17. Lau, H. C. P. *et al.* Inferences of mantle viscosity based on ice age data sets: Radial structure. *JGR Solid Earth* **121**, 6991–7012 (2016).
18. King, S. D. Reconciling laboratory and observational models of mantle rheology in geodynamic modelling. *Journal of Geodynamics* **100**, 33–50 (2016).
19. Steinberger, B. & Calderwood, A. R. Models of large-scale viscous flow in the Earth's mantle with constraints from mineral physics and surface observations. *Geophysical Journal International* **167**, 1461–1481 (2006).
20. Mitrovica, J. X. & Forte, A. M. A new inference of mantle viscosity based upon joint inversion of convection and glacial isostatic adjustment data. *Earth and Planetary Science Letters* **225**, 177–189 (2004).
21. Čížková, H., Van Den Berg, A. P., Spakman, W. & Matyska, C. The viscosity of Earth's lower mantle inferred from sinking speed of subducted lithosphere. *Physics of the Earth and Planetary Interiors* **200–201**, 56–62 (2012).
22. Edmund, E. *et al.* The Thermal Conductivity of Bridgmanite at Lower Mantle Conditions Using a Multi-Technique Approach. *JGR Solid Earth* **129**, e2024JB028823 (2024).
23. Hsieh, W.-P., Deschamps, F., Okuchi, T. & Lin, J.-F. Effects of iron on the lattice thermal conductivity of Earth's deep mantle and implications for mantle dynamics. *Proc. Natl. Acad. Sci. U.S.A.* **115**, 4099–4104 (2018).
24. Manthilake, G. M., De Koker, N., Frost, D. J. & McCammon, C. A. Lattice thermal conductivity of lower mantle minerals and heat flux from Earth's core. *Proc. Natl. Acad. Sci. U.S.A.* **108**, 17901–17904 (2011).

25. Goncharov, A. F., Beck, P., Struzhkin, V. V., Haugen, B. D. & Jacobsen, S. D. Thermal conductivity of lower-mantle minerals. *Physics of the Earth and Planetary Interiors* **174**, 24–32 (2009).

26. Fei, H. *et al.* Variation in bridgmanite grain size accounts for the mid-mantle viscosity jump. *Nature* **620**, 794–799 (2023).

27. Marquardt, H. & Miyagi, L. Slab stagnation in the shallow lower mantle linked to an increase in mantle viscosity. *Nature Geosci* **8**, 311–314 (2015).

28. Hirose, K. Phase transitions in pyrolitic mantle around 670-km depth: Implications for upwelling of plumes from the lower mantle. *J. Geophys. Res.* **107**, (2002).

29. Karato, S. & Wu, P. Rheology of the Upper Mantle: A Synthesis. *Science* **260**, 771–778 (1993).

30. Turcotte, D. L. *Geodynamics*. (Cambridge University Press, West Nyack, 2014).

31. Ranalli, G. *Rheology of the Earth*. (Chapman & Hall, London ; New York, 1995).

32. Clauser, C. & Huenges, E. Thermal Conductivity of Rocks and Minerals. in *Rock Physics and Phase Relations* 105–126 (American Geophysical Union, Washington, D.C., 1995).

33. Gale, A., Dalton, C. A., Langmuir, C. H., Su, Y. & Schilling, J. The mean composition of ocean ridge basalts. *Geochem Geophys Geosyst* **14**, 489–518 (2013).

34. Laporte, D., Toplis, M. J., Seyler, M. & Devidal, J.-L. A new experimental technique for extracting liquids from peridotite at very low degrees of melting: application to partial melting of depleted peridotite. *Contributions to Mineralogy and Petrology* **146**, 463–484 (2004).

35. Hirose, K. & Kushiro, I. Partial melting of dry peridotites at high pressures:

Determination of compositions of melts segregated from peridotite using aggregates of diamond. *Earth and Planetary Science Letters* **114**, 477–489 (1993).

36. Workman, R. K. & Hart, S. R. Major and trace element composition of the depleted MORB mantle (DMM). *Earth and Planetary Science Letters* **231**, 53–72 (2005).

37. Holland, T. J. B., Hudson, N. F. C., Powell, R. & Harte, B. New Thermodynamic Models and Calculated Phase Equilibria in NCFMAS for Basic and Ultrabasic Compositions through the Transition Zone into the Uppermost Lower Mantle. *Journal of Petrology* **54**, 1901–1920 (2013).

38. Jennings, E. S. & Holland, T. J. B. A Simple Thermodynamic Model for Melting of Peridotite in the System NCFMASOCr. *Journal of Petrology* **56**, 869–892 (2015).

39. Holland, T. J. B. & Powell, R. An internally consistent thermodynamic data set for phases of petrological interest. *Journal Metamorphic Geology* **16**, 309–343 (1998).

40. Newton, R. C., Wood, B. J. & Kleppa, O. J. Thermochemistry of silicate solid solutions. *bulmi* **104**, 162–171 (1981).

41. Holland, T. J. B., Green, E. C. R. & Powell, R. Melting of Peridotites through to Granites: A Simple Thermodynamic Model in the System KNCFMASHTOCr. *Journal of Petrology* **59**, 881–900 (2018).
