# Supplementary material for: Sluggish post-garnet transformation controls slab stagnation at the uppermost lower mantle
Source: Nat Commun. 2026 Apr 23;17:5647. doi: 10.1038/s41467-026-72495-5 (PMC13315703; doi:10.1038/s41467-026-72495-5)
Supplement: Supplementary file 2 — Transparent Peer Review file [file 41467_2026_72495_MOESM2_ESM.pdf]

nature portfolio

Peer Review File

Sluggish post-garnet transformation controls slab stagnation at the uppermost lower mantle

Corresponding Author: Dr Jianfeng Yang

This file contains all reviewer reports in order by version, followed by all author rebuttals in order by version.

Attachments originally included by the reviewers as part of their assessment can be found at the end of this file.

Version 0:

Reviewer comments:

Reviewer #1

(Remarks to the Author)
This paper examined effects of sluggish kinetics of the post-garnet transitions in a subducting slab on slab stagnation around 1000 km depth. The authors combined thermodynamic modeling with previous experimental results to demonstrate slab dynamics in the lower mantle. They evaluated detailed situations of subducting slabs by changing important parameters such as dipping angle, and thermal age. They carefully considered situations to simulate slab stagnation and their discussion seem reasonable. I generally recommend publication of this study in Nature Communications after they consider my minor comments.

1. Line 37-50
The authors reviewed previous studies on discussion of the 1000-km depth stagnation. Another mechanism can be discussed: vacancy in bridgmanite (e.g. Liu et al. 2017). To complete this paragraph, I suggest to discuss this mechanism here.
Reference
Liu, Z., Ishii, T., & Katsura, T. (2017). Rapid decrease of MgAlO2.5 component in bridgmanite with pressure. Geochemical Perspectives Letters, 5, 12-18.

2. Line 82-85 (Model set up)
It is better to show the detailed equilibrium phase relations of each slab-constituent rocks: volume fraction of each minerals. Especially volume fractions of garnet are key to understand the conclusion of this paper.

3. Figure S1
The authors used relatively old and inaccurate phase boundaries based on quench experiments. In general, in-situ experimental data were used to discuss mantle dynamics.
I suggest to use the following transition boundaries.
Coe-Stv: Ono et al. (2017). Precise determination of the phase boundary between coesite and stishovite in SiO2. Physics of the Earth and Planetary Interiors, 264, 1-6.
Ol-Wd: Morishima, et al. (1994). The phase boundary between α-and β-Mg2SiO4 determined by in situ X-ray observation. Science, 265(5176), 1202-1203.
Rw-Brg+Per: Chanyshev et al. (2022). Depressed 660-km discontinuity caused by akimotoite–bridgmanite transition. Nature, 601(7891), 69-73.
In addition, how about the effects of wadsleyite to ringwoodite transition and akimotoite-bridgmanite transition?
Especially, akimotoite-bridgmanite transition was suggested to make the 660-km stagnation due to its steep negative slope (Chanyshev et al. 2022).

4. Related to the comment #3, how much these phase transitions assist the 1000-km stagnation? Please discuss the impact of other phase transitions.

5. Sluggish kinetics of olivine ringwoodite transformation is also an important factor to make 660-km stagnation. Can metastable olivine also survives in a 1000-km stagnant slab?

6. Water in minerals can promote their transformations. Can the authors discuss possible presence of water on the 1000-km stagnation?

Reviewer #2

(Remarks to the Author)
This manuscript by Shen et al. addresses the long-standing puzzle of why subducted slabs often stagnate near 1000 km depth in the uppermost lower mantle, despite the absence of a major equilibrium phase transition at that depth in a pyrolitic mantle. Previous explanations invoking chemical heterogeneity or mid-mantle viscosity jumps remain debated, particularly as recent studies suggest the upper lower mantle is largely homogeneous. The authors propose that sluggish kinetics of the post-garnet transformation allow metastable garnet to persist to depths of ~950–1000 km within cold slabs, generating a temporary density deficit that reduces slab buoyancy and induces stagnation. To test this, they employ 2D thermomechanical models incorporating experimentally constrained garnet transformation kinetics and realistic slab compositions. The models systematically explore the effects of garnet grain size, content, slab dip, thermal age, Clapeyron slope, and mantle viscosity jumps.
The results show that under equilibrium conditions, slabs readily descend through the uppermost lower mantle. In contrast, delayed garnet transformation produces transient stagnation at 850–1000 km for durations of 25–80 Myr. The stagnation behavior depends most strongly on garnet grain size, garnet content, and slab dip angle, with steeper slabs more likely to penetrate and shallower slabs more prone to stagnation. Alternative mechanisms, such as a viscosity jump or a negative Clapeyron slope, fail to reproduce observed slab behavior without invoking metastable garnet.
Finally, comparisons with global seismic observations confirm that the modeled slab behavior and stagnation timescales broadly agree with observed slab images and depth-age relations. The study concludes that sluggish post-garnet transformation kinetics play a key role in controlling slab stagnation near 1000 km, offering a geophysically consistent explanation without requiring chemical layering or major rheological stratification.

In my view, the modeling results are straightforward and easy to follow, though the details of the geodynamic setup and parameter choices should be carefully evaluated by experts in geodynamic modeling. While the authors present an intriguing mechanism to explain slab stagnation, I am not convinced that this explanation is either unique or demonstrably superior to other plausible hypotheses. Numerous previous studies, particularly those by Ballmer and colleagues, have demonstrated that factors such as compositional heterogeneity, phase transitions under hydrous or dry conditions, and slab geometry can all significantly influence slab behavior in both the mantle transition zone and the uppermost lower mantle. Geodynamic models incorporating reasonable assumptions — such as basaltic enrichment — have successfully reproduced slab stagnation at similar depths.

The authors largely dismiss these alternatives by citing studies that favor a predominantly pyrolitic lower mantle composition (e.g., Criniti et al., 2021; Kurnosov et al., 2017). However, it is worth noting that the data presented by Kurnosov et al. have since been shown to be problematic. Furthermore, this line of reasoning does not rule out the presence of chemical heterogeneities in the transition zone and uppermost lower mantle — a topic that remains actively debated. Recent seismic and mineral physics studies (e.g., Yu et al., 2023, Science Advances; Gréaux et al., 2019, Nature) provide strong evidence for compositional heterogeneity in this region. In summary, while the authors propose a plausible and interesting mechanism, other explanations remain viable and should not be dismissed prematurely. A more balanced discussion acknowledging these competing hypotheses would strengthen the manuscript.

The second issue concerns the core assumption of sluggish post-garnet transformation kinetics. Although proposed by Nishi et al. (2012), this idea has not been widely accepted. Later experimental work, including Ishii et al. (2023, Nature Geoscience), found no evidence of such metastability even at low temperatures. More critically, even if metastable garnet phases persist for a few hours in laboratory experiments, extrapolating this behavior to geological timescales of tens of millions of years is difficult to justify. Additionally, the assumption that garnet in all lithologies follows the same post-garnet transformation boundary, using parameters derived from basaltic garnet, is a substantial oversimplification. In nature, garnet compositions differ markedly between oceanic crust, harzburgitic, and lherzolitic mantle lithologies, with variations in majorite, pyrope, grossular, and almandine content. These differences could affect both the phase transition pressure-temperature conditions and transformation kinetics. The authors should either test the sensitivity of their model outcomes to this assumption or explicitly acknowledge this source of uncertainty.

Third, while the authors show that garnet grain size exerts a first-order control on slab stagnation behavior, natural constraints on garnet grain size in subducted slabs at lower mantle conditions are very limited. The authors adopt a 1–10 mm range but assume no significant grain size evolution during subduction. Given the critical role of grain size in phase transformation kinetics, potential grain growth or reduction due to deformation or thermal effects should be discussed, along with possible implications for metastability duration and slab behavior.

Fourth, the authors dismiss the potential influence of ferropericlase-related viscosity increases in the mid-lower mantle. However, some recent studies suggest that ferropericlase may contribute to local viscosity contrasts or bulk mantle rheology under certain conditions. Excluding this factor should be better justified, ideally with supporting sensitivity tests or a more detailed discussion of its relevance based on experimental and theoretical constraints.

Lastly, the study relies entirely on 2D thermomechanical models. While these models capture the first-order behavior of subduction, subduction dynamics and mantle convection are inherently 3D processes. Critical features such as slab

rollback, trench retreat, and toroidal flow around slab edges cannot be reproduced in 2D models. The authors should clarify to what extent their 2D results can approximate 3D behavior and whether any key conclusions might be sensitive to dimensionality. A brief discussion or justification of this limitation would improve the manuscript.

Reviewer #3

(Remarks to the Author)
See uploaded Review file

Version 1:

Reviewer comments:

Reviewer #1

(Remarks to the Author)
The authors carefully examined the reviewer comments and responded to reviewer's concerns properly in general. However, I have one more request: they provided mineral fractions only up to 10 GPa. What I expected is to see mineralogy down to 1000 km depth in the case of 1000-km stagnation. Please provide this so that readers can easily understand their assumption on mineralogy.

Reviewer #2

(Remarks to the Author)
I have carefully reviewed the authors' detailed rebuttal and the revised manuscript. The authors have clearly made a significant effort to address the comments from all reviewers. Many of the textual revisions improve the clarity, and several of the newly added analyses—particularly the additional tests on transition-zone phase boundaries and the expanded discussion of uncertainties—strengthen the manuscript. That said, several of the core scientific issues raised in my initial review remain only partially resolved, and I find some of the authors' justifications still not entirely convincing.

Whilst the revised Introduction and Discussion are more balanced than in the original submission, the manuscript still tends to cast the proposed kinetic mechanism as the primary or dominant explanation for slab stagnation near 1000 km. The authors now acknowledge that alternative mechanisms—such as compositional heterogeneity, hydrous phase transitions, and slab geometry—are plausible and supported by prior modelling and seismic studies. However, the treatment of these alternatives remains rather brief, and some statements continue to imply that compositional effects should produce permanent stagnation, which is not consistently supported by the literature. The discussion still underrepresents the breadth of existing evidence for chemical heterogeneity in the transition zone and uppermost lower mantle. A more explicit acknowledgment that multiple mechanisms remain viable, and that their model provides one possible explanation rather than a unique one, would improve the scientific balance of the manuscript.

Furthermore, the premise of the proposed mechanism—sluggish post-garnet transformation—remains more speculative than the authors' response suggests. The authors argue that their assumptions are consistent with the kinetics reported in Kubo et al. (2002, 2008) and that the findings of Ishii et al. (2023) do not contradict their model. However, the published conclusions of Ishii et al. clearly state that sluggish metastability was not observed under the experimental conditions, even at low temperatures. The brief personal communication cited in the rebuttal does not fully resolve this discrepancy. Moreover, extrapolating episodes of partial metastability lasting hours in high-pressure experiments to tens of millions of years in Earth's mantle remains highly uncertain. Whilst the authors correctly note that JMAK-type extrapolation is standard practice, the metastability of majorite- or garnet-related reactions is less well constrained than that of olivine, and the manuscript would benefit from a more cautious and transparent treatment of this uncertainty.

Thirdly, although the authors provide several arguments for using basaltic garnet as a proxy for the garnet transformation boundary across all slab lithologies, this remains a substantial simplification. The different garnet compositions in oceanic crust, harzburgite, and fertile mantle—particularly variations in majorite, pyrope, grossular, and almandine—can influence both equilibrium boundaries and reaction kinetics. The fact that grossular and almandine may remain metastable to somewhat higher pressures does not necessarily imply that their kinetic behaviours mimic basaltic garnet. The authors' sensitivity tests on total garnet abundance help partially address this uncertainty, but they do not fully capture compositional effects on kinetics. A more explicit acknowledgment of this limitation in the Discussion is needed.

The authors' treatment of grain size is generally acceptable. Their parameter sweep includes a reasonable range, and the revised text now acknowledges the possibility of grain-size evolution. However, the manuscript still lacks a discussion of grain-size reduction associated with deformation, which may be as important as grain growth. This omission does not fundamentally undermine the results but should be recognised as an additional source of uncertainty.

Regarding ferropericlase-related viscosity effects, the authors now present a more convincing argument in the revised manuscript by incorporating a sensitivity test and referencing recent experimental studies. Although debate remains about lower-mantle rheology, the authors' response is adequate and improves clarity.

Finally, the new discussion of 2D versus 3D modelling limitations is helpful and clarifies why the authors believe their main conclusions remain robust despite dimensional simplification. Whilst 3D effects such as slab rollback and toroidal flow are

not captured, the authors now clearly state that their proposed mechanism relates primarily to vertical buoyancy forces within wide subduction zones, where 2D approximations are more defensible.

On the whole, the authors have strengthened the manuscript, but several key assumptions—particularly those concerning garnet kinetics and compositional simplifications—still require more cautious framing. I would recommend that the authors further temper their conclusions and make clearer that their mechanism represents one plausible explanation among several competing hypotheses. With these refinements, the manuscript would make for a more balanced and scientifically robust contribution to the ongoing discussion of slab behaviour in the uppermost lower mantle.

Reviewer #3

(Remarks to the Author)

Version 2:

Reviewer comments:

Reviewer #2

(Remarks to the Author)
The authors have clearly made a substantial effort to respond to the reviewers, and the tone of their rebuttal is professional and constructive. Several of the revisions are meaningful. In particular, the rephrasing of the Abstract and Discussion to frame the sluggish post-garnet transformation as “an alternative explanation” rather than the dominant or unique mechanism is a genuine improvement. The addition of sensitivity tests, expanded uncertainty discussion, and clarification of the 2D modeling limitations all strengthen the manuscript compared to the original submission. The new supplementary mineralogical profiles down to ~40 GPa also improve transparency regarding their phase assemblage assumptions.

That said, the core scientific concern remains only partially resolved: the entire geodynamic conclusion hinges on the long-term metastability of garnet in the uppermost lower mantle. The authors now acknowledge uncertainty more explicitly, but the physical plausibility of metastable garnet surviving over geological timescales remains weakly constrained. Their response to the experimental tension with Ishii et al. (2023) is still somewhat selective. While it is true that Ishii et al. focused on equilibrium phase boundaries, the fact remains that sluggish metastability was not reported even at low temperature conditions. The authors’ reliance on Kubo et al. kinetics and standard JMAK extrapolation is methodologically defensible in principle, but the extrapolation spans many orders of magnitude in time and is applied to a reaction system far less experimentally constrained than the olivine–spinel transition. In the revised manuscript they acknowledge this as a “key uncertainty,” but scientifically, this is not a secondary uncertainty—it is foundational to the proposed mechanism. The model quantifies consequences if sluggish kinetics operate, but whether they realistically do so remains speculative.

A second unresolved issue concerns compositional simplification. The authors now explicitly admit that using basaltic garnet kinetics across all lithologies is a simplification driven by limited data, and they have added a discussion of this limitation. This improves transparency. However, the sensitivity tests on total garnet abundance do not fully address kinetic variability among pyrope-rich, grossular-rich, or almandine-bearing garnets. Since reaction kinetics can be strongly composition-dependent, this assumption could significantly affect the magnitude and even existence of metastable buoyancy anomalies. The revision properly acknowledges this uncertainty, but the mechanistic robustness under realistic compositional diversity remains insufficiently demonstrated.

The grain-size treatment is now more balanced, and the added discussion of deformation-induced grain-size reduction is appropriate. This does not critically undermine the model but reinforces that kinetic delay is sensitive to poorly constrained microphysical evolution. Similarly, the ferropericlase rheology discussion and the dimensional (2D vs 3D) limitations are adequately handled in the revised version. These aspects are no longer major weaknesses.

The broader interpretational balance has improved, but the manuscript still walks a fine line between proposing a plausible hypothesis and implying explanatory sufficiency. The authors state that their mechanism may operate independently or synergistically with compositional heterogeneity and viscosity contrasts. However, the model does not quantitatively compare the relative strength of these mechanisms. Thus, the study demonstrates that sluggish kinetics could generate stagnation under certain assumptions, but it does not demonstrate that this mechanism is required, dominant, or even equally competitive with existing explanations. As long as this limitation is clearly understood, the contribution is conceptual rather than definitive.

Overall, the manuscript is now more cautious and better framed, but its central premise remains experimentally underconstrained. Whether this is acceptable depends on the journal’s threshold for speculative yet testable geodynamic hypotheses. The study does not appear technically flawed, although it rests on a kinetic assumption that is plausible but not strongly validated. In my assessment, the manuscript is suitable for publication provided that the key kinetic assumption is considered to fall within a reasonable range of physically plausible scenarios.

Open Access This Peer Review File is licensed under a Creative Commons Attribution 4.0 International License, which permits use, sharing, adaptation, distribution and reproduction in any medium or format, as long as you give appropriate credit to the original author(s) and the source, provide a link to the Creative Commons license, and indicate if changes were made.
In cases where reviewers are anonymous, credit should be given to 'Anonymous Referee' and the source.
The images or other third party material in this Peer Review File are included in the article's Creative Commons license, unless indicated otherwise in a credit line to the material. If material is not included in the article's Creative Commons license and your intended use is not permitted by statutory regulation or exceeds the permitted use, you will need to obtain permission directly from the copyright holder.
To view a copy of this license, visit https://creativecommons.org/licenses/by/4.0/

Authors' Response to the Reviewers' Comments

Journal: Nature Communications

Manuscript #: NCOMMS-25-28454

Tile of the paper: Sluggish post-garnet transformation controls slab stagnation at the uppermost lower mantle

Authors: Yongqiang Shen, Jianfeng Yang, Liang Zhao

Dear reviewers,

Thanks for your constructive comments which have significantly improved the quality and clarity of our manuscript. Please see our point-by-point response to your comments below. Our response in this letter is labeled in blue, and the changes in the main text are highlighted in red. The line numbers correspond to the revised manuscript.

REVIEWER COMMENTS

Reviewer #1 (Remarks to the Author):

This paper examined effects of sluggish kinetics of the post-garnet transitions in a subducting slab on slab stagnation around 1000 km depth. The authors combined thermodynamic modeling with previous experimental results to demonstrate slab dynamics in the lower mantle. They evaluated detailed situations of subducting slabs by changing important parameters such as dipping angle, and thermal age. They carefully considered situations to simulate slab stagnation and their discussion seem reasonable. I generally recommend publication of this study in Nature Communications after they consider my minor comments.

We thank you the reviewer for the positive evaluation and valuable suggestions.

1. Line 37-50

The authors reviewed previous studies on discussion of the 1000-km depth stagnation. Another mechanism can be discussed: vacancy in bridgmanite (e.g. Liu et al. 2017). To complete this paragraph, I suggest to discuss this mechanism here.

Reference

Liu, Z., Ishii, T., & Katsura, T. (2017). Rapid decrease of MgAlO2.5 component in bridgmanite with pressure. Geochemical Perspectives Letters, 5, 12-18.

This is a relevant paper suggesting the rapid decrease in oxygen vacancies in

bridgmanite with depth leads to increased viscosity that may cause slab stagnation at ~1000 km. Therefore, we have cited it to support the evidence of viscosity jump in the Introduction and Discussion.

2. Line 82-85 (Model set up)

It is better to show the detailed equilibrium phase relations of each slab-constituent rocks: volume fraction of each minerals. Especially volume fractions of garnet are key to understand the conclusion of this paper.

We thank the reviewer for the suggestion. The mineral phase proportions for different chemical compositions are calculated by using Perple_X, shown in Fig. S5. We have also updated it to overlie the modelled temperature-pressure ranges. The initial volume fraction of garnet content for each rock type is explicitly mentioned in Line 81 and is now referred to Fig. S5 as well.

3. Figure S1

The authors used relatively old and inaccurate phase boundaries based on quench experiments. In general, in-situ experimental data were used to discuss mantle dynamics.

I suggest to use the following transition boundaries.

Coe-Stv: Ono et al. (2017). Precise determination of the phase boundary between coesite and stishovite in SiO_2. Physics of the Earth and Planetary Interiors, 264, 1-6.

Ol-Wd: Morishima, et al. (1994). The phase boundary between α-and β-Mg_2SiO_4 determined by in situ X-ray observation. Science, 265(5176), 1202-1203.

Rw-Brg+Per: Chanyshev et al. (2022). Depressed 660-km discontinuity caused by akimotoite–bridgmanite transition. Nature, 601(7891), 69-73.

In addition, how about the effects of wadsleyite to ringwoodite transition and akimotoite-bridgmanite transition?

Especially, akimotoite-bridgmanite transition was suggested to make the 660-km stagnation due to its steep negative slope (Chanyshev et al. 2022).

We thank the reviewer for the suggestions regarding the use of updated phase boundaries and for the discussion on akimotoite-bridgmanite transition.

We have updated the phase boundaries as you suggest in our Fig. S1. Furthermore, we have performed additional model tests to investigate the influence of these phase boundaries near the mantle transition zone on our proposed mechanism. All the model results are displayed in Fig. S12 and are also described in the main text (Lines 144-158).

The key finding from these new results is that the akimotoite-bridgmanite transition has little effect, although its Clapeyron slope is steeply negative, on lower mantle slab

penetration and stagnation at ~1000 km, due to the low content of akimotoite in the subducted slab. Furthermore, the new tests (Fig. S12) confirm that the kinetics of the post-garnet transformation remain the dominant control on slab behavior in the uppermost lower mantle, even when more complex phase transitions in the mantle transition zone are considered.

We thank your guidance to strengthen the mineral physics and the overall robustness of our study.

4. Related to the comment #3, how much these phase transitions assist the 1000-km stagnation? Please discuss the impact of other phase transitions.

We thank the reviewer for the follow-up question. As mentioned in the reply to comment #3, we conduct model tests exploring separately how these transition boundaries affect slab behavior at the uppermost lower mantle (Fig. S12). Please see the reply above, as well as the description in the main text (Lines 144-158).

We found that the phase transition of ringwoodite-bridgmanite has the largest influence among these phase transitions. With Clapeyron slope varying from -0.1 to -3.0 MPa/K, the slab stagnation mode transits from penetration mode to 660-km-stagnation mode, consistent with previous studies. Although phase transitions in the mantle transition zone could affect the stagnation behavior of the subducting slab, slab stagnation at the uppermost lower mantle could not be achieved without metastable garnet.

5. Sluggish kinetics of olivine ringwoodite transformation is also an important factor to make 660-km stagnation. Can metastable olivine also survives in a 1000-km stagnant slab?

We agree with the reviewer that the olivine-ringwoodite kinetics may be an important factor for slab stagnation at the mantle transition zone. This is also confirmed by previous numerical models (e.g., Schmeling et al., 1999; Tetzlaff & Schmeling, 2000). In this scenario, the stagnant slab above the 660-km-discontinuity could be heated up by surrounding mantle which promotes the olivine transformation (e.g., Mosenfelder et al., 2001). From our reference model, the temperature of the slab core is ~1000-1100°C which is beyond the temperature stability (~700°C, Mosenfelder et al., 2001) of the metastable olivine. Therefore, we suggest that the metastable olivine has little or no effect on the 1000-km slab stagnation.

6. Water in minerals can promote their transformations. Can the authors discuss possible presence of water on the 1000-km stagnation?

We thank the reviewer for this comment which allows us to elaborate on the implications of our model for slabs with different hydration states. Due to the effect of water on the transformation, we can speculate two end-member cases: a dry vs. a hydrous slab. (1) In a severely dehydrated slab (i.e., dry slab), the transformation kinetics would be expected to be slower than in our models. This would enhance the metastability of garnet, potentially leading to more prolonged and pronounced stagnation at the 850–1000 km depths. (2) Conversely, a water-rich slab would potentially promote the transformation, leading to shorter stagnation or even uninterrupted penetration.

We have added the discussion in the main text (Lines 229-236).

Reviewer #2 (Remarks to the Author):

This manuscript by Shen et al. addresses the long-standing puzzle of why subducted slabs often stagnate near 1000 km depth in the uppermost lower mantle, despite the absence of a major equilibrium phase transition at that depth in a pyrolitic mantle. Previous explanations invoking chemical heterogeneity or mid-mantle viscosity jumps remain debated, particularly as recent studies suggest the upper lower mantle is largely homogeneous. The authors propose that sluggish kinetics of the post-garnet transformation allow metastable garnet to persist to depths of ~950–1000 km within cold slabs, generating a temporary density deficit that reduces slab buoyancy and induces stagnation. To test this, they employ 2D thermomechanical models incorporating experimentally constrained garnet transformation kinetics and realistic slab compositions. The models systematically explore the effects of garnet grain size, content, slab dip, thermal age, Clapeyron slope, and mantle viscosity jumps.
The results show that under equilibrium conditions, slabs readily descend through the uppermost lower mantle. In contrast, delayed garnet transformation produces transient stagnation at 850–1000 km for durations of 25–80 Myr. The stagnation behavior depends most strongly on garnet grain size, garnet content, and slab dip angle, with steeper slabs more likely to penetrate and shallower slabs more prone to stagnation. Alternative mechanisms, such as a viscosity jump or a negative Clapeyron slope, fail to reproduce observed slab behavior without invoking metastable garnet.
Finally, comparisons with global seismic observations confirm that the modeled slab behavior and stagnation timescales broadly agree with observed slab images and depth-age relations. The study concludes that sluggish post-garnet transformation kinetics

play a key role in controlling slab stagnation near 1000 km, offering a geophysically consistent explanation without requiring chemical layering or major rheological stratification.

We thank the reviewer for your constructive comments and helpful suggestions.

In my view, the modeling results are straightforward and easy to follow, though the details of the geodynamic setup and parameter choices should be carefully evaluated by experts in geodynamic modeling. While the authors present an intriguing mechanism to explain slab stagnation, I am not convinced that this explanation is either unique or demonstrably superior to other plausible hypotheses. Numerous previous studies, particularly those by Ballmer and colleagues, have demonstrated that factors such as compositional heterogeneity, phase transitions under hydrous or dry conditions, and slab geometry can all significantly influence slab behavior in both the mantle transition zone and the uppermost lower mantle. Geodynamic models incorporating reasonable assumptions — such as basaltic enrichment — have successfully reproduced slab stagnation at similar depths.

The authors largely dismiss these alternatives by citing studies that favor a predominantly pyrolitic lower mantle composition (e.g., Criniti et al., 2021; Kurnosov et al., 2017). However, it is worth noting that the data presented by Kurnosov et al. have since been shown to be problematic. Furthermore, this line of reasoning does not rule out the presence of chemical heterogeneities in the transition zone and uppermost lower mantle — a topic that remains actively debated. Recent seismic and mineral physics studies (e.g., Yu et al., 2023, Science Advances; Gréaux et al., 2019, Nature) provide strong evidence for compositional heterogeneity in this region. In summary, while the authors propose a plausible and interesting mechanism, other explanations remain viable and should not be dismissed prematurely. A more balanced discussion acknowledging these competing hypotheses would strengthen the manuscript.

We thank the reviewer for the critical comment and for emphasizing the importance of a balanced discussion regarding alternative hypotheses for slab stagnation. We agree that the composition of the lower mantle, particularly the uppermost lower mantle region, is an area of active research and debate, and that our original manuscript may have presented our kinetic mechanism as overly exclusive. Our primary goal is not to definitively rule out all forms of compositional heterogeneity but to demonstrate that slab stagnation at ~1000 km can occur robustly in a pyrolitic mantle due to a previously under-explored kinetic effect. We believe this is a significant finding because it provides a mechanism that is consistent with many geophysical and mineral physics data supporting an average pyrolitic bulk lower mantle, without requiring a fundamental, large-scale change in mantle composition.

Specifically, our model emphasizes that the delayed phase transition of garnet produces transient slab stagnation, which aligns well with seismic observations that many slabs appear to sink continuously to greater depths without prolonged arrest in the uppermost lower mantle (e.g., the Sunda & Nazca slabs). A purely compositional barrier might be expected to cause more permanent ponding (and further influence the whole mantle convection), which is not universally observed.

On the other hand, we also acknowledge the value and plausibility of previous studies/hypotheses that our new mechanism is complementary rather than exclusive. Therefore, we have revised the Introduction (Lines 39-45) and Discussion (Lines 196-216) sections. We have also removed the reference Kurnosov et al. (2017).

The second issue concerns the core assumption of sluggish post-garnet transformation kinetics. Although proposed by Nishi et al. (2012), this idea has not been widely accepted. Later experimental work, including Ishii et al. (2023, Nature Geoscience), found no evidence of such metastability even at low temperatures. More critically, even if metastable garnet phases persist for a few hours in laboratory experiments, extrapolating this behavior to geological timescales of tens of millions of years is difficult to justify. Additionally, the assumption that garnet in all lithologies follows the same post-garnet transformation boundary, using parameters derived from basaltic garnet, is a substantial oversimplification. In nature, garnet compositions differ markedly between oceanic crust, harzburgitic, and lherzolitic mantle lithologies, with variations in majorite, pyrope, grossular, and almandine content. These differences could affect both the phase transition pressure-temperature conditions and transformation kinetics. The authors should either test the sensitivity of their model outcomes to this assumption or explicitly acknowledge this source of uncertainty.

We thank the reviewer for raising the critical points regarding the kinetic assumptions in our model. We agree that these are important considerations and below are the key items to your comments:

(1) **Assumption of sluggish post-garnet transformation.** We agree that the kinetics of mantle phase transitions are complex processes. However, the foundation of our model is based on a body of experimental work that has consistently observed sluggish kinetics for the post-garnet transformation. The kinetic parameters and the functional form of the growth law employed in this study is mainly based on the experimental results of Kubo et al. (2002, 2008), which has been widely cited and accepted in the community. We further added the insights of Nishi et al. (2012) by implementing a pressure threshold for completion of the transformation, accounting for the intracrystalline growth mechanism.

Crucially, regarding the more recent study by Ishii et al. (2023) cited by the reviewer, we have consulted directly with the lead author, Dr. Takayuki Ishii. He has confirmed that in their experiments "*The reaction was very slow and the complete reaction did not occur during the experiments, suggesting this transformation is sluggish. But this always occurs with any transformations of minerals because experiments were always near phase boundary, where transformation rate is very slow in general…*" Therefore, rather than contradicting our model, their experimental results somehow support our assumption.

(2) **Extrapolation to geological timescale.** We agree this is a critical consideration. The extrapolation of mineral growth data in laboratory experiment to geological timescales is based on the John-Mehl-Avrami-Kolmogorov (JMAK) equation. This equation describes how solids transform from one phase to another and the kinetics of crystallization. Cahn (1957) examined this theory and developed it to grain boundary nucleated reactions. This theory was then used in kinetics study of mineral phase transition in the deep mantle. One of the typical findings is the sluggish transformation of olivine to spinel in low temperature (e.g., Rubie and Ross II, 1994). This slow transformation could form a metastable olivine wedge in the subducted slab, which is widely confirmed by seismic studies. The extrapolation procedure used in our study is similar with that in olivine-spinel transformation study. Therefore, while extrapolation always carries uncertainty, our method follows a standard and validated procedure in mineral physics and geodynamics. The fact that our model produces stagnation timescales that are geologically reasonable and consistent with seismic observations provides a compelling consistency check for this approach.

(3) **Simplification of garnet composition and kinetics.** We agree with the reviewer that the phase boundaries and the content of different garnet compositions in each slab are different. This simplification is mainly made based on following consideration. 1) The basaltic crust has the largest garnet content among different slab components at the lower mantle conditions. The post-garnet transition boundary of the basaltic crust is close to that those of pyrope and majorite in pyrolitic mantle at uppermost lower mantle conditions. 2) The phase boundary of majorite in harzburgite is close to that of ringwoodite-bridgmanite transition (Fig. S1b) and its effect on density could be integrated with that of ringwoodite-bridgmanite transition. 3) For grossular and almandine, although they decompose at ~19-21 and 22-26 GPa in equilibrium respectively, high pressure experiments suggested slow kinetics of their decomposition reaction, which allow them to survive in metastable phases to pressures of 27-30 GPa, overlapping with the basaltic garnet transition zone. Therefore, using the basaltic crust boundary as a proxy is a reasonable simplification that captures the essential physics.

Crucially, our model's sensitivity tests on total garnet content (Fig. 5) effectively account for the first-order uncertainty stemming from compositional variation. Additionally, we explicitly explained these assumptions/uncertainties in the Methods section (Lines 347-367 and 511-522).

Third, while the authors show that garnet grain size exerts a first-order control on slab stagnation behavior, natural constraints on garnet grain size in subducted slabs at lower mantle conditions are very limited. The authors adopt a 1–10 mm range but assume no significant grain size evolution during subduction. Given the critical role of grain size in phase transformation kinetics**,** potential grain growth or reduction due to deformation or thermal effects should be discussed, along with possible implications for metastability duration and slab behavior.
We thank the reviewer for raising the point regarding the natural constraints on garnet grain size. We agree with the reviewer that grain size may evolve during subduction and its initial value is uncertain. Precisely because of this uncertainty, we designed our study to systematically test a wide range of plausible grain sizes (2, 4, and 6 mm), representative of the 1–10 mm range observed in natural samples and experimental studies. Our model results demonstrate that the mechanism of kinetic stagnation is a robust feature across this entire parameter range. While the specific depth and duration of stagnation are modulated by grain size, the fundamental phenomenon occurs in all tested scenarios. This indicates that our central conclusion is not dependent on this poorly constrained parameter but is valid for a wide range of probable conditions. Finally, we have added the discussion to explicitly acknowledge the potential for grain size evolution in the main text (Lines 221-229).

Fourth, the authors dismiss the potential influence of ferropericlase-related viscosity increases in the mid-lower mantle. However, some recent studies suggest that ferropericlase may contribute to local viscosity contrasts or bulk mantle rheology under certain conditions. Excluding this factor should be better justified, ideally with supporting sensitivity tests or a more detailed discussion of its relevance based on experimental and theoretical constraints.
We thanks the reviewer for the comment to better justify the ferropericlase-related viscosity. In this study, we do not consider the influence of ferropericlase-related viscosity increases in the mid-lower mantle, since dynamic dislocation modeling suggested the bridgmanite deforms more rapidly than periclase by pure climb creep under natural strain rate (Cordier et al., 2023). This suggested that the bulk rheology of the lower mantle is governed by bridgmanite. Furthermore, we explicitly tested a scenario with a significant mid-mantle viscosity increase (Fig. S11) as proposed by Fei

et al. (2023) for bridgmanite. In our penetration models, the calculated viscosity in the slab's vicinity actually exceeds the value proposed by Marquardt and Miyagi (2015) for a ferropericlase-induced jump. Despite this, slabs in our models continued to penetrate, demonstrating that even a strong viscosity increase is insufficient to cause stagnation when overridden by the localized deformation around the slab.

Therefore, while we acknowledge ferropericlase's role in mantle rheology, our results show that a viscosity jump alone may be not a primary control on stagnation in our models. We have detailed description (Lines 133-143) and discussion (Lines 209-220) in the main text.

Lastly, the study relies entirely on 2D thermomechanical models. While these models capture the first-order behavior of subduction, subduction dynamics and mantle convection are inherently 3D processes. Critical features such as slab rollback, trench retreat, and toroidal flow around slab edges cannot be reproduced in 2D models. The authors should clarify to what extent their 2D results can approximate 3D behavior and whether any key conclusions might be sensitive to dimensionality. A brief discussion or justification of this limitation would improve the manuscript.

We thank the reviewer for the comment regarding the limitations of 2D modeling. We agree with the reviewer that subduction in nature is an inherently 3D process, and features like toroidal flow around slab edges cannot be reproduced in 2D models.

However, the core physical mechanism we investigate—the kinetically-induced buoyancy of a metastable phase—is a first-order, intrinsic property of the slab. The balance of forces that governs stagnation is fundamentally a vertical process that is robustly captured in our 2D models.

Notably, our 2D models are specifically relevant to the geological examples we cite. Many of the key seismic observations of stagnation at ~1000 km depth (e.g., beneath the Sunda, Peru, and Kermadec subduction zones) are located in the middle section of wide subduction zones, where flow is predominantly poloidal (2D-like) and the effects of toroidal flow at slab edges are minimized. For these regions, our 2D models provide a good approximation.

We also acknowledge that near the slab edge such as the Kamchatka subduction zone, 3D effects like toroidal flow and enhanced slab retreat may be dominant. These processes may lead to a shallower slab dip, which our own model tests identify as a primary control favoring shallower stagnation (e.g., at 660 km or 850 km). Thus, while the full 3D complexity is not captured in 2D models, our parameter study of dip angle provides hints on the slab behavior expected at slab edges.

Ultimately, while our 2D study can well establish the kinetic mechanism as a primary control, we acknowledge that future 3D models are needed to fully resolve how edge-driven flow influences the spatial pattern of stagnation, but we believe that the fundamental process we identify is expected to be widely applicable. We have added further discussion in the main text (Lines 236-250).

Reviewer 3:

This manuscript explores the dynamical efffects of transition-kinetic delay in the post-garnet phase transition in the earths mantle. Such delay could explain the stagnation and flattening of subducting lithospheric slabs as observed in images of seismic tomography. Slab stagnation was originally explained by the negative Clapeyron slope of the ringwoodite to bridgemanite+ferropericlse transition in combination with a visocosity increase nearr 660 km depth. The current work focusses on stagnation at larger depth that is not well explained by the ringwoodite dissociation. Alternative explanations for these larger depths have a.o. been a possible rheological transition in the range 660-1000km, for example in (Fei et al., Nature, 2023), (Pokorny et al., Earth and Planetary Science Letters, 2023). This alternative explanantion is contested by the present authors who argue for a different rheological model of the lower mantle. The authors present extensive modelling results of the subduction of a lithospherice slab. The results show that the parametrized kinetic delay of the post-garnet phase transition does have a stagnating effect o0n the slab in the depth range > 660 km that can explain the images from seismic tomography. The presented work is an important contribution to mantle dynamics, reconciling observational data from seismolgy and mineral physics and I recommend accepting the manuscript for publication, after taking into account following comments.

Thank you for the summarize and encouraging comments.

Comments on the manuscript: - In the methods section the authors state that they have used the Boussineq approximation in their numerical mantle convection model. Since they have included non-Boussinesq terms for adiabatic heating, viscous dissipation and latent heat in their energy equation the model is better referred to as ’extended Boussinesq’.

Thank you for pointing it out. We have corrected the “Boussineq approximation” as “extended Boussineq approximation” (Line 321).

- The extended Boussinesq model assumes a divergence free velocity field for an incompressible medium. At the same time the equation of state contains the linearized effect of finite compressibility and the supporting moaterial, Fig. S6, indicates that the density variation does not fit a model with small variation around a uniform value. This should be further explained in the method- and discussion sections.

We thank the reviewer for the constructive comment and for highlighting the need for greater clarity regarding the used Stokes equation.

Indeed, the formulation used in our study follows the Extended Boussinesq Approximation (EBA). The EBA retains the incompressible mass conservation equation (divergence-free) while incorporating key non-Boussinesq effects in the energy equation (adiabatic, shear, and latent heating). We acknowledge this is a simplification, and we have added a paragraph in the Methods section to justify the choice.

In practice, the EBA neglects the dynamic volume changes associated with advection and compression/expansion but can still account for the buoyancy forces arising from thermal and compositional anomalies relative to the background state. In our study, the primary mechanism for slab stagnation at ~1000 km is the density deficit caused by metastable garnet, a buoyancy effect that is fully captured within the EBA framework. The dominant errors associated with the EBA, as quantified by Gassmöller et al. (2020), arise from neglecting volumetric changes during heating/cooling or phase transitions. Since our main finding is independent of the volume effects, the EBA is a suitable and computationally efficient approximation. Furthermore, the error introduced by the EBA (or related approximation) is small compared to other model uncertainties according to Gassmöller et al. (2020). The more sophisticated approach proposed by them is probably crucial for problems such as localized dehydration reactions, but may not be necessary to capture the first-order buoyancy-driven stagnation mechanism we identified in this study.

For clarity, we have added an explanation at Lines 336-344.

- Textual suggestions:
line 218: 'core-mantle boundary' => 'core-mantle boundary region'

We have modified it according to your suggestion.

line 284: 'technique to' => 'technique is applied to'

We have modified it according to your suggestion.

- Missing reference

Murakami et al., 2024

The relevant text has been removed, and this reference is also deleted.

- Superfluous repetition of references:
  - Ishii et al., 2023
  - Kubo et al., 2002
  - Nishi et al., 2012
  - Reali etal., 2019
  - Van Mierlo et al., 2013

We have deleted the repeated references.

References in this letter

Schmeling, H., Monz, R. & Rubie, D. C. The influence of olivine metastability on the dynamics of subduction. Earth and Planetary Science Letters 165, 55–66 (1999).

Tetzlaff, M. & Schmeling, H. The influence of olivine metastability on deep subduction of oceanic lithosphere. Physics of the Earth and Planetary Interiors 120, 29–38 (2000).

Mosenfelder, J. L., Marton, F. C., Ross, C. R., Kerschhofer, L. & Rubie, D. C. Experimental constraints on the depth of olivine metastability in subducting lithosphere. Physics of the Earth and Planetary Interiors 127, 165–180 (2001).

Kubo, T. et al. Metastable garnet in oceanic crust at the top of the lower mantle. Nature 420, 803–806 (2002).

Kubo, T. et al. Kinetics of the post-garnet transformation: Implications for density and rheology of subducting slabs. Physics of the Earth and Planetary Interiors 170, 181–192 (2008).

Nishi, M., Irifune, T., Ohfuji, H. & Tange, Y. Intracrystalline nucleation during the post-garnet transformation under large overpressure conditions in deep subducting slabs. Geophysical Research Letters 39, 2012GL053915 (2012).

Cahn, J. W. The kinetics of grain boundary nucleated reactions. Acta Metallurgica 4, 449–459 (1956).

Rubie, D. C. & Ross, C. R. Kinetics of the olivine-spinel transformation in subducting lithosphere: experimental constraints and implications for deep slab processes. Physics of the Earth and Planetary Interiors 86, 223–243 (1994).

Cordier, P. et al. Periclase deforms more slowly than bridgmanite under mantle conditions. Nature 613, 303–307 (2023).

Marquardt, H. & Miyagi, L. Slab stagnation in the shallow lower mantle linked to an increase in mantle viscosity. Nature Geosci 8, 311–314 (2015).

Gassmöller, R., Dannberg, J., Bangerth, W., Heister, T. & Myhill, R. On formulations of compressible mantle convection. Geophysical Journal International 221, 1264–1280 (2020).

Authors’ Response to the Reviewers’ Comments

Journal: Nature Communications
Manuscript #: NCOMMS-25-28454A
Tile of the paper: Sluggish post-garnet transformation controls slab stagnation at the uppermost lower mantle
Authors: Yongqiang Shen, Jianfeng Yang, Liang Zhao

Dear reviewers,
Thanks again for your constructive comments and please see our point-by-point response to your comments below. Our response in this letter is labeled in blue, and the changes in the main text are highlighted in red. The line numbers and figures correspond to the revised manuscript.

Reviewer #1 (Remarks to the Author):

The authors carefully examined the reviewer comments and responded to reviewer's concerns properly in general.
However, I have one more request: they provided mineral fractions only up to 10 GPa. What I expected is to see mineralogy down to 1000 km depth in the case of 1000-km stagnation. Please provide this so that readers can easily understand their assumption on mineralogy.
We thank the reviewer for the positive feedback and for this final suggestion.
We have now added a new figure to the Supplementary Information (Figure S5) showing the calculated mineral phase proportions for each slab component (harzburgite, pyrolite, lherzolite, and basalt) extending to 40 GPa (~1000 km depth). To approximate the non-equilibrium conditions associated with a sluggish post-garnet transition, we excluded the relevant product phases (e.g., aluminum-bearing bridgmanite) in the calculation. This approach allows garnet to persist metastably into the uppermost lower mantle, consistent with our model assumption. Please refer to Figure S5 and its caption for a detailed presentation and explanation of the results.

Reviewer #2 (Remarks to the Author):

I have carefully reviewed the authors' detailed rebuttal and the revised manuscript. The authors have clearly made a significant effort to address the comments from all reviewers. Many of the textual revisions improve the clarity, and several of the newly added analyses—particularly the additional tests on transition-zone phase boundaries and the expanded discussion of uncertainties—strengthen the manuscript. That said, several of the core scientific issues raised in my initial review remain only partially resolved, and I find some of the authors' justifications still not entirely convincing.

We thank the reviewer for the careful and constructive assessment of our revised manuscript, and for acknowledging this significant effort and improvements made. We appreciate your engagement and will now address the remaining points below to further strengthen the balance and clarity of the manuscript.

Whilst the revised Introduction and Discussion are more balanced than in the original submission, the manuscript still tends to cast the proposed kinetic mechanism as the primary or dominant explanation for slab stagnation near 1000 km. The authors now acknowledge that alternative mechanisms—such as compositional heterogeneity, hydrous phase transitions, and slab geometry—are plausible and supported by prior modelling and seismic studies. However, the treatment of these alternatives remains rather brief, and some statements continue to imply that compositional effects should produce permanent stagnation, which is not consistently supported by the literature. The discussion still underrepresents the breadth of existing evidence for chemical heterogeneity in the transition zone and uppermost lower mantle. A more explicit acknowledgment that multiple mechanisms remain viable, and that their model provides one possible explanation rather than a unique one, would improve the scientific balance of the manuscript.

We thank the reviewer for this important suggestion to improve the balance of our manuscript. We agree that multiple mechanisms may contribute to slab stagnation at ~1000 km depth, and that presenting our kinetic mechanism as one contributor within a spectrum of viable hypotheses strengthens the discussion.

In response, we have refocused our discussion to present our kinetic model as one plausible mechanism among other viable explanations, such as compositional heterogeneity and mantle viscosity jumps. We have explicitly acknowledged the evidence supporting these alternatives and removed the comparative text regarding permanent versus transient stagnation (see Lines 195-219). For example, we describe that "*…This kinetic mechanism does not exclude the potential roles of chemical heterogeneity or rheological variations. Rather, it could operate independently, or synergistically with them…*"

Moreover, we have also revised our ABSTRACT to emphasize that our proposed mechanism is "*an alternative explanation for slab stagnation...*"

We believe these revisions provide a more balanced and scientifically robust perspective.

Furthermore, the premise of the proposed mechanism—sluggish post-garnet transformation—remains more speculative than the authors' response suggests. The authors argue that their assumptions are consistent with the kinetics reported in Kubo et al. (2002, 2008) and that the findings of Ishii et al. (2023) do not contradict their model. However, the published conclusions of Ishii et al. clearly state that sluggish metastability was not observed under the experimental conditions, even at low temperatures. The brief personal communication cited in the rebuttal does not fully resolve this discrepancy. Moreover, extrapolating episodes of partial metastability lasting hours in high-pressure experiments to tens of millions of years in Earth's mantle remains highly uncertain. Whilst the authors correctly note that JMAK-type extrapolation is standard practice, the metastability of majorite- or garnet-related reactions is less well constrained than that of olivine, and the manuscript would benefit from a more cautious and transparent treatment of this uncertainty.

We thank the reviewer for raising these important points regarding the uncertainties in garnet transformation kinetics. We fully agree that extrapolating laboratory kinetics to geological timescales involves uncertainty, and that the kinetics of the post-garnet transformation are indeed less constrained than those of the olivine-spinel transition. As suggested, we have now incorporated a more explicit and cautious discussion of these fundamental uncertainties in the revised manuscript (see lines 256-268 in the Discussion section).

Regarding the specific point on Ishii et al. (2023), their primary focus was on precisely determining the equilibrium phase boundary, not on directly testing the kinetics metastability. Therefore, their experimental design (e.g., using a three-phase starting mixture to bracket the boundary) was not aimed at observing—or ruling out—long-lived metastable garnet. Our model does not rely upon their studies.

Our study explicitly adopts the kinetic formulation of Kubo et al. (2008), as detailed in the Methods/Supplement (Lines 389-411). Its primary goal is to explore the geodynamic implications if such sluggish kinetics are operative.

We fully agree that extrapolating laboratory-derived rates to geological timescales is fundamental uncertainty in geodynamic modeling. To frame this transparently, we have added text stating that the viability of garnet metastability over millions of years remains a key uncertainty, dependent on future experimental verification. Thus, our model serves to quantify the potential geodynamic consequences of this mechanism and to highlight the critical need for better kinetic constraints in future work. We believe this approach strengthens the manuscript by clearly delineating the assumptions and contributions of our study.

Thirdly, although the authors provide several arguments for using basaltic garnet as a proxy for the garnet transformation boundary across all slab lithologies, this remains a substantial simplification. The different garnet compositions in oceanic crust, harzburgite, and fertile mantle—particularly variations in majorite, pyrope, grossular, and almandine—can influence both equilibrium boundaries and reaction kinetics. The fact that grossular and almandine may remain metastable to somewhat higher pressures does not necessarily imply that their kinetic behaviours mimic basaltic garnet. The authors' sensitivity tests on total garnet abundance help partially address this uncertainty, but they do not fully capture compositional effects on kinetics. A more explicit acknowledgment of this limitation in the Discussion is needed.

We thank the reviewer for the suggestions. We agree that different garnet compositions in oceanic crust, harzburgite, and fertile mantle can influence both equilibrium boundaries and reaction kinetics, and the reaction kinetics of grossular and almandine could be different from that of the pyrope. Our choice was necessitated by the current lack of comprehensive experimental data on the kinetics of decomposition for different garnet solutions.

As the reviewer notes, our systematic sensitivity analysis on total garnet content is designed to address the first-order uncertainty of compositional variation.

We acknowledge that this does not fully capture potential compositional effects on kinetics. Therefore, we have added explicit acknowledgment of these uncertainties in the Discussion (see Line 256-268).

The authors' treatment of grain size is generally acceptable. Their parameter sweep includes a reasonable range, and the revised text now acknowledges the possibility of grain-size evolution. However, the manuscript still lacks a discussion of grain-size reduction associated with deformation, which may be as important as grain growth. This omission does not fundamentally undermine the results but should be recognised as an additional source of uncertainty.

We thank the reviewer for the positive comments. We agree that deformation-induced grain size reduction is a process that could compete with thermal grain growth in a subducting slab, and acknowledging this provides a more complete picture of the uncertainties involved.

We have added the relevant discussion on the effect of the grain-size reduction on kinetics of post-garnet transition and associated slab stagnation process. Please check line 227-230 in Discussion.

Regarding ferropericlase-related viscosity effects, the authors now present a more convincing argument in the revised manuscript by incorporating a sensitivity test and referencing recent experimental studies. Although debate remains about lower-mantle rheology, the authors' response is adequate and improves clarity.

We thank the reviewer for the positive comments. We agree that debate continues regarding lower-mantle rheology, and we are pleased our revision has provided adequate clarity for the context of this study.

Finally, the new discussion of 2D versus 3D modelling limitations is helpful and clarifies why the authors believe their main conclusions remain robust despite dimensional simplification. Whilst 3D effects such as slab rollback and toroidal flow are not captured, the authors now clearly state that their proposed mechanism relates primarily to vertical buoyancy forces within wide subduction zones, where 2D approximations are more defensible.

We thank the reviewer for the positive comments.

On the whole, the authors have strengthened the manuscript, but several key assumptions—particularly those concerning garnet kinetics and compositional simplifications—still require more cautious framing. I would recommend that the authors further temper their conclusions and make clearer that their mechanism represents one plausible explanation among several competing hypotheses. With these refinements, the manuscript would make for a more balanced and scientifically robust contribution to the ongoing discussion of slab behaviour in the uppermost lower mantle.

We sincerely thank the reviewer for the comprehensive and constructive engagement throughout the reviewer process. The comments have significantly strengthened our manuscript.

We fully accept the final recommendation. We have undertaken a careful review of the manuscript to ensure our conclusions are tempered and presented with appropriate caution.

We have attributed our mechanism as one plausible explanation that may operate with other mechanisms. We have also thoroughly acknowledged associated uncertainties.

We hope the revised manuscript will provide more balanced and scientifically robust perspective advocated for to the field.

Reviewer #3

Comment on revised manuscript: Sluggish post-garnet transformation controls slab stagnation at the uppermost lower mantle

Authors: Yongqiang Shen, Jianfeng Yang, Liang Zhao

The authors have repsonded properly to my comments of August 15 on the original version of the manuscript.

I recommend accepting the revised manuscript for publication.

We thank the reviewer's helpful comments again.

Authors' Response to the Reviewers' Comments

Journal: Nature Communications
Manuscript #: NCOMMS-25-28454B
Tile of the paper: Sluggish post-garnet transformation controls slab stagnation at the uppermost lower mantle
Authors: Yongqiang Shen, Jianfeng Yang, Liang Zhao

Dear reviewers,
Thanks again for your constructive comments and please see our point-by-point response to your comments below. Our response in this letter is labeled in blue, and the changes in the main text are also highlighted in blue. The line numbers and figures correspond to the revised manuscript.

Reviewer #2 (Remarks to the Author):

The authors have clearly made a substantial effort to respond to the reviewers, and the tone of their rebuttal is professional and constructive. Several of the revisions are meaningful. In particular, the rephrasing of the Abstract and Discussion to frame the sluggish post-garnet transformation as "an alternative explanation" rather than the dominant or unique mechanism is a genuine improvement. The addition of sensitivity tests, expanded uncertainty discussion, and clarification of the 2D modeling limitations all strengthen the manuscript compared to the original submission. The new supplementary mineralogical profiles down to ~40 GPa also improve transparency regarding their phase assemblage assumptions.

We thank the reviewer for acknowledging the substantial improvements, including the rephrasing, sensitivity tests, and expanded discussion of uncertainties.

That said, the core scientific concern remains only partially resolved: the entire geodynamic conclusion hinges on the long-term metastability of garnet in the uppermost lower mantle. The authors now acknowledge uncertainty more explicitly, but the physical plausibility of metastable garnet surviving over geological timescales remains weakly constrained. Their response to the experimental tension with Ishii et al. (2023) is still somewhat selective. While it is true that Ishii et al. focused on equilibrium phase boundaries, the fact remains that sluggish metastability was not reported even at low temperature conditions. The authors' reliance on Kubo et al. kinetics and standard JMAK extrapolation is methodologically defensible in principle, but the extrapolation spans many orders of magnitude in time and is applied to a reaction system far less experimentally constrained than the olivine–spinel transition. In the revised manuscript they acknowledge this as a "key uncertainty," but scientifically, this is not a secondary uncertainty—it is foundational to the proposed mechanism. The model quantifies consequences if sluggish kinetics operate, but whether they realistically do so remains speculative.

We thank the reviewer for raising this point. We fully agree that the long-term metastability of garnet remains less experimentally constrained, and that extrapolating laboratory kinetics to geological timescales carries significant uncertainty. In the revised manuscript, we have modified the expression "key premise" to "foundational premise" in Lines 257-258. As the reviewer notes, further high-pressure, low-temperature experimental studies are needed to better constrain the kinetics of the post-garnet transformation. We now highlight this need in Lines 268–270, following the acknowledgment of uncertainties. We view our study as a hypothesis that quantifies the potential geodynamic consequences if such sluggish kinetics operate, thereby motivating future experimental work.

A second unresolved issue concerns compositional simplification. The authors now explicitly admit that using basaltic garnet kinetics across all lithologies is a simplification driven by limited data, and they have added a discussion of this limitation. This improves transparency. However, the sensitivity

tests on total garnet abundance do not fully address kinetic variability among pyrope-rich, grossular-rich, or almandine-bearing garnets. Since reaction kinetics can be strongly composition-dependent, this assumption could significantly affect the magnitude and even existence of metastable buoyancy anomalies. The revision properly acknowledges this uncertainty, but the mechanistic robustness under realistic compositional diversity remains insufficiently demonstrated.

We thank the reviewer for this comment. We acknowledge that using basaltic garnet kinetics across all lithologies is a simplification, and that garnet composition (e.g., pyrope, grossular, almandine) likely influences transformation kinetics. In our manuscript, we have explicitly discussed this limitation (Line 259-267). While our sensitivity tests on total garnet abundance partially address the role of garnet content, we agree that compositional effects on kinetics cannot be fully captured without further experimental data. We highlight the need of high-pressure experiments to clarify the effect of garnet composition in Lines 268–270.

The broader interpretational balance has improved, but the manuscript still walks a fine line between proposing a plausible hypothesis and implying explanatory sufficiency. The authors state that their mechanism may operate independently or synergistically with compositional heterogeneity and viscosity contrasts. However, the model does not quantitatively compare the relative strength of these mechanisms. Thus, the study demonstrates that sluggish kinetics could generate stagnation under certain assumptions, but it does not demonstrate that this mechanism is required, dominant, or even equally competitive with existing explanations. As long as this limitation is clearly understood, the contribution is conceptual rather than definitive.

We thank the reviewer for acknowledging the improvements made to the grain-size treatment and the discussion of deformation-induced grain-size reduction. We are pleased that these aspects are now considered adequate.

Overall, the manuscript is now more cautious and better framed, but its central premise remains experimentally underconstrained. Whether this is acceptable depends on the journal's threshold for speculative yet testable geodynamic hypotheses. The study does not appear technically flawed, although it rests on a kinetic assumption that is plausible but not strongly validated. In my assessment, the manuscript is suitable for publication provided that the key kinetic assumption is considered to fall within a reasonable range of physically plausible scenarios.

We thank the reviewer for the thoughtful assessment and for recognizing the improvements made in the revised manuscript. We greatly appreciate the constructive engagement throughout the review process, which has helped us refine the framing and clarity of our work. We are grateful for the reviewer's time and expertise.

Review of: Sluggish post-garnet transformation controls slab stagnation
at the uppermost lower mantle
Authors: Yongqiang Shen, Jianfeng Yang, Liang Zhao

This manuscript explores the dynamical efffects of transition-kinetic delay in the post-garnet phase transition in the earths mantle.
Such delay could explain the stagnation and flattening of subducting lithospheric slabs as observed in images of seismic tomography.
Slab stagnation was originally explained by the negative Clapeyron slope of the ringwoodite to bridgemanite+ferropericlse transition in combination with a visocosity increase nearr 660 km depth.

The current work focusses on stagnation at larger depth that is not well explained by the ringwoodite dissociation.

Alternative explanations for these larger depths have a.o. been a possible rheological transition in the range 660-1000km, for example in (Fei et al., Nature, 2023), (Pokorny et al., Earth and Planetary Science Letters, 2023).
This alternative explanantion is contested by the present authors who argue for a different rheological model of the lower mantle.

The authors present extensive modelling results of the subduction of a lithospherice slab.
The results show that the parametrized kinetic delay of the post-garnet phase transition does have a stagnating effect o0n the slab in the depth range > 660 km that can explain the images from seismic tomography.

The presented work is an important contribution to mantle dynamics, reconciling observational data from seismolgy and mineral physics and I recommend accepting the manuscript for publication, after taking into account following comments.

Comments on the manuscript:
- In the methods section the authors state that they have used the Boussineq approximation in their numerical mantle convection model.
  Since they have included non-Boussinesq terms for adiabatic heating, viscous dissipation and latent heat in their energy equation the model is better referred to as 'extended Boussinesq'.

- The extended Boussinesq model assumes a divergence free velocity field for an incompressible medium. At the same time the equation of state contains the linearized effect of finite compressibility and the supporting moaterial, Fig. S6, indicates that the density variation does not fit a model with small variation around a uniform value.
  This should be further explained in the method- and discussion sections.

- Textual suggestions:
  line 218: 'core-mantle boundary' => 'core-mantle boundary region'
  line 284: 'technique to' => 'technique is applied to'

- Missing reference
  Murakami et al., 2024

- Superfluous repetition of references:
  - Ishii et al., 2023
  - Kubo et al., 2002
  - Nishi et al., 2012
  - Reali etal., 2019
  - Van Mierlo et al., 2013

Comment on revised manuscript: Sluggish post-garnet transformation controls slab stagnation at the uppermost lower mantle
Authors: Yongqiang Shen, Jianfeng Yang, Liang Zhao

The authors have repsonded properly to my comments of August 15 on the original version of the manuscript.
I recommend accepting the revised manuscript for publication.
